# Three-dimensional genome landscape of primary human cancers

**DOI:** 10.1038/s41588-025-02188-0

**Published:** 2025-05-12

**Authors:** Kathryn E. Yost, Yanding Zhao, King L. Hung, Kaiyuan Zhu, Duo Xu, M. Ryan Corces, Shadi Shams, Bryan H. Louie, Shahab Sarmashghi, Laksshman Sundaram, Jens Luebeck, Stanley Clarke, Ashley S. Doane, Jeffrey M. Granja, Hani Choudhry, Marcin Imieliński, Andrew D. Cherniack, Ekta Khurana, Vineet Bafna, Ina Felau, Jean C. Zenklusen, Peter W. Laird, Christina Curtis, Cancer Genome Atlas Analysis Network, Kathryn E. Yost, Yanding Zhao, King L. Hung, Kaiyuan Zhu, Duo Xu, M. Ryan Corces, Shahab Sarmashghi, Laksshman Sundaram, Jens Luebeck, Ashley S. Doane, Jeffrey M. Granja, Andrew D. Cherniack, Ekta Khurana, Vineet Bafna, Ina Felau, Jean C. Zenklusen, Peter W. Laird, Christina Curtis, William J. Greenleaf, Howard Y. Chang, William J. Greenleaf, Howard Y. Chang

**Affiliations:** 1 Center for Personal Dynamic Regulomes, Stanford University School of Medicine, Stanford, CA USA; 2 Department of Dermatology, Stanford University School of Medicine, Stanford, CA USA; 3 Department of Genetics, Stanford University School of Medicine, Stanford, CA USA; 4 Department of Computer Science and Engineering, University of California, San Diego, La Jolla, CA USA; 5 Sandra and Edward Meyer Cancer Center, Weill Cornell Medicine, New York City, NY USA; 6 Department of Physiology and Biophysics, Weill Cornell Medicine, New York City, NY USA; 7 Gladstone Institute of Neurological Disease, San Francisco, CA USA; 8 Gladstone Institute of Data Science and Biotechnology, San Francisco, CA USA; 9 Department of Neurology, University of California, San Francisco, San Francisco, CA USA; 10 Broad Institute of MIT and Harvard, Cambridge, MA USA; 11 Department of Computer Science, Stanford University, Stanford, CA USA; 12 Illumina AI laboratory, Illumina Inc, Foster City, CA USA; 13 NVIDIA Bio Research, NVIDIA, Santa Clara, CA USA; 14 Vilcek Institute of Graduate Biomedical Sciences, NYU Grossman School of Medicine, New York City, NY USA; 15 Laura and Isaac Perlmutter Cancer Center, New York University Langone Health, New York City, NY USA; 16 Department of Pathology, New York University Langone Health, New York City, NY USA; 17 New York Genome Center, New York City, NY USA; 18 Department of Pathology and Laboratory Medicine, Weill Cornell Medicine, New York City, NY USA; 19 Department of Biochemistry, Faculty of Science, Cancer and Mutagenesis Unit, King Fahd Center for Medical Research, King Abdulaziz University, Jeddah, Saudi Arabia; 20 Department of Medical Oncology, Dana–Farber Cancer Institute, Boston, MA USA; 21 Harvard Medical School, Boston, MA USA; 22 Institute for Computational Biomedicine, Weill Cornell Medicine, New York City, NY USA; 23 Englander Institute for Precision Medicine, Weill Cornell Medicine, New York City, NY USA; 24 National Cancer Institute, NIH, Bethesda, MD USA; 25 Center for Epigenetics, Van Andel Research Institute, Grand Rapids, MI USA; 26 Department of Medicine, Stanford University School of Medicine, Stanford, CA USA; 27 Chan Zuckerberg Biohub, San Francisco, CA USA; 28 Department of Applied Physics, Stanford University, Stanford, CA USA; 29 Howard Hughes Medical Institute, Stanford University School of Medicine, Stanford, CA USA; 30 Present Address: Pathos AI, Chicago, IL USA; 31 Present Address: Whitehead Institute for Biomedical Research, Cambridge, MA USA

**Keywords:** Cancer, Gene regulation, Epigenomics

## Abstract

Genome conformation underlies transcriptional regulation by distal enhancers, and genomic rearrangements in cancer can alter critical regulatory interactions. Here we profiled the three-dimensional genome architecture and enhancer connectome of 69 tumor samples spanning 15 primary human cancer types from The Cancer Genome Atlas. We discovered the following three archetypes of enhancer usage for over 100 oncogenes across human cancers: static, selective gain or dynamic rewiring. Integrative analyses revealed the enhancer landscape of noncancer cells in the tumor microenvironment for genes related to immune escape. Deep whole-genome sequencing and enhancer connectome mapping provided accurate detection and validation of diverse structural variants across cancer genomes and revealed distinct enhancer rewiring consequences from noncoding point mutations, genomic inversions, translocations and focal amplifications. Extrachromosomal DNA promoted more extensive enhancer rewiring among several types of focal amplification mechanisms. These results suggest a systematic approach to understanding genome topology in cancer etiology and therapy.

## Abstract (web summary)

This study characterizes the three-dimensional (3D) genome architecture of 15 primary human cancer types from The Cancer Genome Atlas. The analyses identify different archetypes of enhancer usage and enhancer rewiring events due to different classes of mutations and structural variants.

## Main

In every human cell, 2 m of DNA is extensively folded within a ~10-µm nucleus. Eukaryotic genomes are hierarchically organized in three dimensions to enable transcriptional regulation by distal *cis*-regulatory elements. Chromosomes are subdivided into multimegabase (Mb) A and B compartments, which interact in a homotypic fashion and are enriched for euchromatin versus heterochromatin, respectively^1^. Megabase-sized topologically associating domains (TADs) facilitate DNA interactions within TADs but generally exclude interactions between TADs^2–6^. Enhancer–promoter (E–P) loops connect distal enhancers to target genes located 10–100 kilobases away, enabling cell-type-specific gene expression^7–9^. These three scales of genome architecture can operate independently^10^. Alterations in gene expression, DNA methylation and chromatin accessibility are widespread in primary human cancers^11,12^. However, functionally linking *cis*-regulatory elements to target genes remains challenging due to regulatory element redundancy, cell-type-specific activity and large genomic distances between *cis*-regulatory elements and their target genes^13^. While prior studies have illustrated the potential impact of altered chromosome topology on enhancer rewiring in cancer^14^, a systematic understanding of the three-dimensional (3D) architecture of cancer genomes is still lacking. Differences in 3D genome organization between cell line models and primary tissue^15^ as well as patient-specific genetic alterations highlight the importance of chromosome conformation profiling in primary cancer samples.

Cancer genomes are characterized by frequent structural variations (SVs) that have the potential to alter 3D genome organization, enabling interactions with otherwise distant regulatory elements^16,17^. In addition to simple SVs, including duplications, deletions, inversions and translocations^17^, ongoing genomic instability in cancer can lead to complex structures through chromothripsis, breakage-bridge fusion events and extrachromosomal DNA (ecDNA) formation. ecDNAs are ~100-kb- to 5-Mb-sized circular DNA molecules that enable massive oncogene expression and lead to poor patient outcome^18^. SVs can lead to alterations in both gene copy number (CN) and DNA element connectivity, but the functional consequences on gene regulation are poorly understood^19,20^. Chromosome conformation has emerged as a powerful tool to assemble and characterize SVs^21^. Mapping the 3D cancer genome may clarify the structure of SVs as well as determine their functional consequences on gene regulation.

Here we map the enhancer connectome of primary human cancers by HiChIP, a protein-directed chromosome conformation method, to simultaneously assess enhancer activity measured by histone H3 lysine 27 acetylation (H3K27ac) and interactions with target loci^22,23^, two features that are jointly predictive of gene expression^24^. We leverage and integrate multidimensional data from The Cancer Genome Atlas (TCGA), including expanded deep whole-genome sequencing (WGS) and single-cell chromatin accessibility mapping with 3D genome architecture, to address the role of chromosome topology in cancer gene regulation.

## Results

### Multiple scales of 3D genome organization in human cancers

**Fig. 1:**
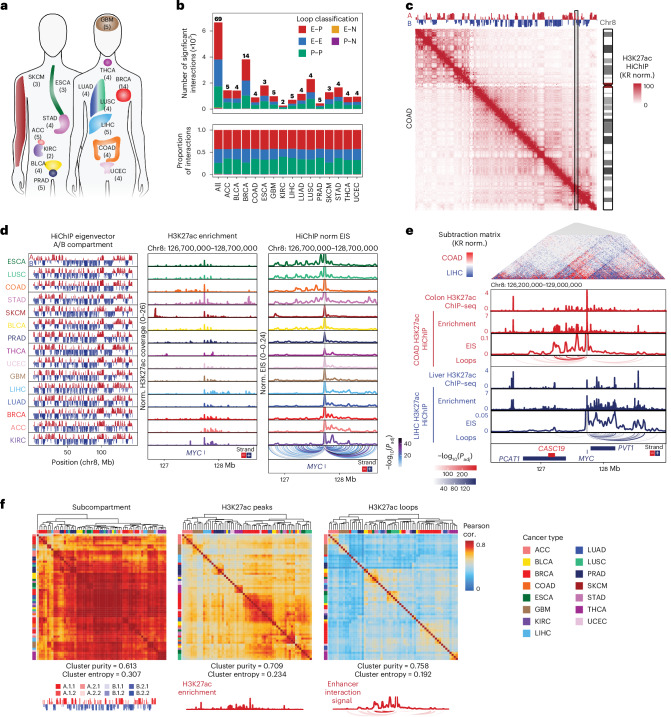
HiChIP identifies high-resolution chromosome conformation in primary human cancers across multiple scales. **a**, Schematic representation of the 15 cancer types profiled in this study. **b**, Stacked bar plot of the number of unique significant FitHiChIP interactions identified by H3K27ac HiChIP by cancer type and colored by loop classification (E–P, E–E, P–P, E–N and P–N). The numbers shown above each bar represent the number of samples profiled for each cancer type. **c**, KR matrix balancing-normalized H3K27ac HiChIP contact matrix at 250-kb resolution for merged COAD samples on chromosome 8. Top track displays the first principal component of Pearson’s matrix eigenvector of the KR-normalized observed/expected matrix, corresponding to A/B compartment. **d**, First eigenvector of the KR-normalized observed/expected matrix, corresponding to A/B compartment, for all samples merged by cancer type (left). One-dimensional H3K27ac signal enrichment at the *MYC* locus normalized by reads overlapping TSS for all samples merged by cancer type (middle). Interaction profiles of the *MYC* promoter representing EIS for all samples merged by cancer type (right). Significant loop interactions colored by adjusted *P* value are shown below. *P* values were calculated using a two-sided binomial test and corrected using the BH procedure. Cancer types are ordered based on H3K27ac signal bias at the *MYC* locus. **e**, Subtraction matrix comparing KR-normalized H3K27ac HiChIP at 10-kb resolution from merged COAD and LIHC samples at the *MYC* locus (top). Tracks visualize H3K27ac ChIP–seq enrichment from normal tissue profiled by ENCODE, HiChIP 1D H3K27ac enrichment, interaction profiles of the *MYC* promoter, and significant loop interactions colored by adjusted *P* value. *P* values were calculated using a two-sided binomial test and corrected using the BH procedure. **f**, Unsupervised hierarchical clustering of vectorized HiChIP subcompartment annotations (left), HiChIP 1D H3K27ac signal (middle), and HiChIP 2D interaction signal (right). Heatmap colored by Pearson correlation coefficients. Cluster purity quantifies the degree that samples of the same cancer type cluster together with higher values, indicating better clustering performance, while for cluster entropy, lower values indicate better clustering performance. Representative subcompartments, H3K27ac enrichment and EIS tracks illustrating the data type used for correlation analysis are shown at bottom.

**Extended Data Fig. 1:**
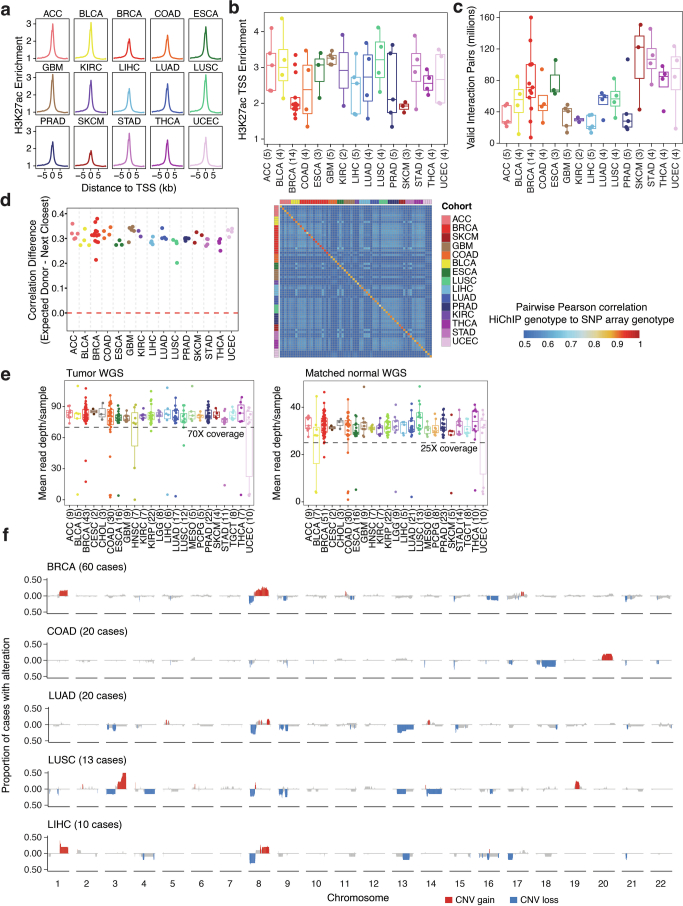
Quality control of H3K27ac HiChIP and WGS data. **a**, Enrichment of HiChIP 1D H3K27ac signal at transcription start sites for all samples merged by cancer type. H3K27ac enrichment per base pair at regions ±2000 bp from the transcription start site is normalized to the number of insertions between ±1900–2000 bp from the transcription start site. **b**, Box plot of the transcription start site enrichment values for all samples of each cancer type. Number of samples from different donors listed for each cancer type. Box centerline, median; box limits, upper and lower quartiles; box whiskers, 1.5× interquartile range. **c**, Box plot of the total valid interaction pairs for all samples of each cancer type. Number of samples from different donors listed for each cancer type. Box centerline, median; box limits, upper and lower quartiles; box whiskers, 1.5× interquartile range. **d**, Genotype correlations between HiChIP genotype and SNP array-derived genotype. Correlation with the next closest match is derived from correlating with all other 69 donors profiled by SNP array by TCGA. Samples that match their expected donor better than all other donors have a correlation difference value above zero (red line, left). Heatmap showing the pairwise Pearson correlation between HiChIP genotype and SNP array genotype, with high correlation along the diagonal indicating HiChIP sample genotypes are most highly correlated with the expected donor genotype based on SNP array (right). **e**, Box plot of the mean read depth per sample for tumor WGS and matched normal WGS. Dashed lines indicate targeted coverage of 70× for tumor WGS and 25× for matched normal WGS. Number of samples from different donors listed for each cancer type. Box centerline, median; box limits, upper and lower quartiles; box whiskers, 1.5× interquartile range. **f**, Genome-wide frequencies of copy-number alterations (CNVs) identified by WGS quantified as proportion of cases with CNV gain (log_2_(CNV) > 1) or CNV loss (log_2_(CNV) < −1) in 1 Mb genomic windows. Identified CNV alterations are consistent with prior findings, such as chromosome 8q gain in BRCA and LIHC^86–88^ and chromosome 3q gain in LUSC ^89,90^.

We profiled genome-wide chromosome conformation in 69 tumor samples representing 15 primary human cancer types using H3K27ac HiChIP^22,23^ (Methods). These 15 cancer types were chosen based on overlap with samples previously profiled by the assay of transposase-accessible chromatin using sequencing (ATAC–seq)^12^ and to represent the diversity of human cancers (Fig. 1a and Supplementary Table 1). All HiChIP experiments demonstrated signal enrichment at gene promoters and sufficient numbers of uniquely mapped contacts for further analysis (Extended Data Fig. 1a–c). To enable integration with additional donor-matched data generated by TCGA, including ATAC–seq, RNA sequencing (RNA-seq) and WGS data, we validated donor identity based on single-nucleotide polymorphism (SNP) genotyping calls (Extended Data Fig. 1d)^12^. WGS of 268 TCGA samples analyzed for chromatin accessibility was also extended to 75× coverage for tumor samples and 25× coverage for matched normal samples to facilitate interpretation of CN variations (CNVs), point mutations and SVs (Extended Data Fig. 1e,f and Supplementary Table 2; Methods).

**Extended Data Fig. 2:**
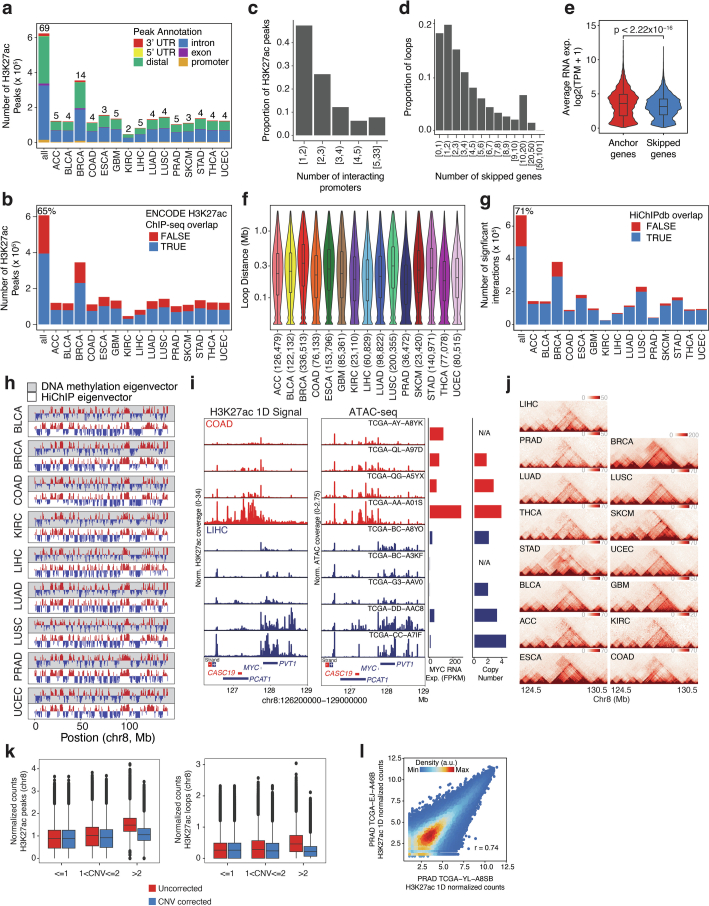
Comparison of HiChIP data with prior epigenomic profiling. **a**, Stacked bar plot of unique H3K27ac 1D peaks by cancer type colored by peak classification. N = number of samples per cancer type. **b**, Stacked bar plot of unique H3K27ac 1D peaks by cancer type colored by overlap with ENCODE H3K27ac ChIP–seq peaks (Supplementary Table 8). **c**, Bar plot of interacting promoters linked to H3K27ac peaks. **d**, Bar plot of genes skipped by HiChIP loops. **e**, Violin with box plots of the average RNA expression of genes at loop anchors (n = 256,888 gene–loop pairs) and skipped genes between loop anchors (n = 218,050 gene–loop pairs). *P* value determined by two-sided Wilcoxon rank-sum test and not adjusted for multiple comparisons. Box centerline, median; box limits, upper and lower quartiles; box whiskers, 1.5× interquartile range. **f**, Violin with box plot of loop distances by cancer type. N = number of loops detected for each cancer type. Box plot components as in (**e**). **g**, Stacked bar plot of unique significant interactions identified by H3K27ac HiChIP by cancer type and colored by overlap with previously identified loops in HiChIPdb^25^. **h**, Comparison of the first eigenvector of the DNA methylation correlation matrix^26^ with the H3K27ac HiChIP eigenvector by cancer type. **i**, Comparison of H3K27ac 1D signal enrichment and bulk ATAC–seq^12^ for individual COAD and LIHC samples at the *MYC* locus (left). Bar plot of *MYC* RNA expression and copy number from WGS (right). **j**, KR-normalized H3K27ac HiChIP contact matrix at the *MYC* locus at 50 kb resolution for all samples merged by cancer type. **k**, Box plots of H3K27ac peak (left) and loop (right) signal before and after copy-number normalization for peaks or loops with relative copy number ≤1 (n = 1,684,034 sample-peak pairs and n = 1,051,956 sample–loop pairs), 1 < CN ≤ 2 (n = 2,384,070 sample-peak pairs and n = 978,152 sample–loop pairs), or >2 (n = 166,180 sample-peak pairs and n = 543,760 sample–loop pairs). Box plot components as in (**e**). **l**, Scatter plot of H3K27ac 1D signal enrichment in the union peak set in two PRAD samples. Each dot represents an individual peak.

We identified 665,682 unique significant interactions, or loops, associated with putative regulatory elements marked by H3K27ac, including complex E–P interactions such as enhancer-skipping of nearest genes (Fig. 1b and Extended Data Fig. 2a–f). Additionally, we compared our pan-cancer loop set with previously identified loops from H3K27ac HiChIP profiling of cell lines and primary tissue samples (Extended Data Fig. 2g)^25^. Overall, 71% of our loops overlapped with previously identified loops, and we also identified 188,887 looping interactions not observed in previous datasets. HiChIP interaction matrices revealed A/B compartment level organization at the megabase scale reflected in the first eigenvector of the correlation matrix, which was largely consistent across different cancer types and concordant with A/B compartments estimated from DNA methylation correlation matrices^26^ (Fig. 1c,d and Extended Data Fig. 2h).

To explore enhancer connectome diversity between different cancer types, we first considered the *MYC* oncogene located on chromosome 8, which is regulated by surrounding tissue-specific enhancers^12,27^. We assessed one-dimensional (1D) H3K27ac ChIP enrichment detected by HiChIP and observed H3K27ac enrichment either at regulatory elements located 5′ of *MYC* in cancer types such as colon adenocarcinoma (COAD) or at 3′ regulatory elements as in liver hepatocellular carcinoma (LIHC; Fig. 1d,e). This bias in H3K27ac reflected tissue-specific H3K27ac enrichment observed in healthy colon and liver, as well as previously observed trends in chromatin accessibility from matched samples^12,28^ (Fig. 1e and Extended Data Fig. 2i). Furthermore, we observed corresponding biases in 3D organization at the *MYC* locus using HiChIP, reflected in differential contact frequency in the interaction matrix and direction of significant loops linked to the *MYC* promoter (Fig. 1e and Extended Data Fig. 2j). Finally, 5′ or 3′ bias in enhancer activity was also reflected in enhancer interaction signal (EIS) at the *MYC* promoter, as determined by virtual 4C analysis, which reflects both H3K27ac ChIP signal strength and chromosome conformation contact strength with the designated anchor (Fig. 1d,e).

**Extended Data Fig. 3:**
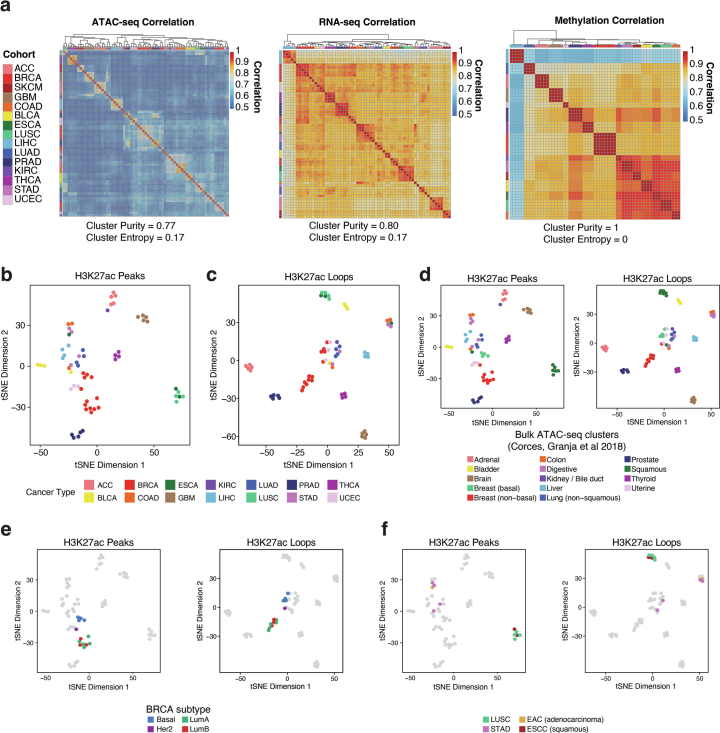
Unsupervised clustering of H3K27ac peaks and HiChIP interactions. **a**, Heatmap showing the unsupervised clustering of ATAC–seq, RNA-seq and DNA methylation array. Heatmap colored by Pearson correlation coefficients. Cluster purity quantifies the degree that samples of the same cancer type cluster together with higher values indicating better clustering performance, while for cluster entropy lower values indicate better clustering performance. **b**, Unsupervised t-SNE on the top 15 principal components for the top 10,000 variable H3K27ac peaks in the union peak set across all cancer types. Each dot represents a unique sample colored by cancer type. **c**, Unsupervised t-SNE on the top 10 principal components for the top 10,000 variable H3K27ac HiChIP loops in the union loop set across all cancer types. Each dot represents a unique sample colored by cancer type. **d**, t-SNE colored by bulk ATAC–seq cluster annotations from ref. ^12^. **e**, t-SNE colored by BRCA subtype^91^. **f**, t-SNE colored by ESCA subtype^33^.

We further examined the scales of genome topology that distinguished human cancer types, leveraging the multiscale data yielded by HiChIP. We noted that H3K27ac enrichment as well as 2D interaction signals were impacted by CNVs, and for subsequent analyses, we applied CN correction based on WGS ploidy-corrected CNV calls, excluding seven samples without matched WGS from further analysis (Extended Data Fig. 2k; Methods). First, we performed Pearson correlation and hierarchical clustering using vectorized subcompartment annotations reflecting higher order chromosome conformation^29^ (Fig. 1f). Individual samples exhibited high pairwise correlation at the subcompartment level, and some cancer types were not well separated by hierarchical clustering, similar to prior observations of conserved compartment organization between different cell and tissue types^1,8,30^. Second, we found that 1D H3K27ac enrichment associated with cell-type-specific enhancers^31,32^ provided better cancer-type specificity, reflected in a higher cluster purity and lower cluster entropy following hierarchical clustering (Fig. 1f and Extended Data Fig. 2l; Methods). Finally, 2D HiChIP signal at significant interactions in the union loop set provided the best separation between different cancer types, and clustering was concordant with prior clustering based on bulk RNA-seq, ATAC–seq and DNA methylation^12^ (Fig. 1f and Extended Data Fig. 3a).

**Extended Data Fig. 4:**
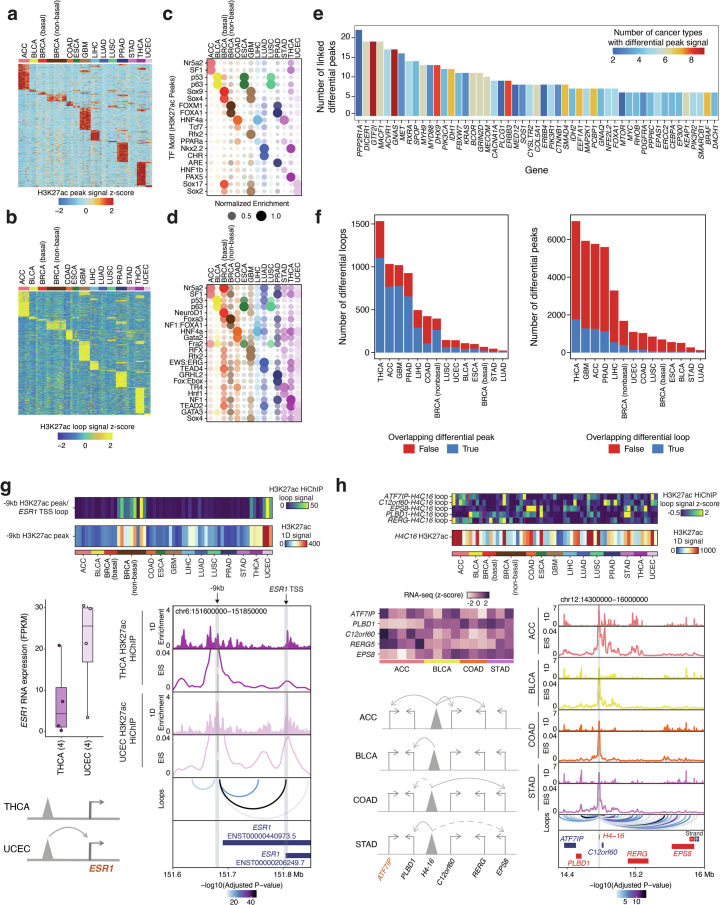
Cancer-type-specific H3K27ac peaks and HiChIP interactions. **a**, Heatmap of H3K27ac enrichment at cancer-type-specific peaks (n = 28,716). **b**, Heatmap of HiChIP contact enrichment at cancer-type-specific loops (n = 5,073). **c**, TF motif enrichment in cancer-type-specific H3K27ac peaks. **d**, TF motif enrichment in cancer-type-specific loops. **e**, Bar plot of linked differential peaks for oncogenes with >5 differential peaks, colored by number of cancer types with differential peaks. **f**, Stacked bar plot of differential loops colored by overlap with differential peaks for each cancer type (left). All differential loops overlap at least one H3K27ac peak. Stacked bar plot of differential peaks colored by overlap with differential loops for each cancer type (right). Only differential peaks overlapping any identified loops were considered (27,166/28,716 differential peaks). **g**, H3K27ac HiChIP signal z scores across samples for the enhancer–promoter (E–P) interaction between *ESR1* promoter and −9 kb H3K27ac peak (top of top panel). H3K27ac 1D signal z scores across samples for −9 kb H3K27ac peak (top of bottom panel). Box plot of *ESR1* RNA expression (n = number of samples from different donors) and schematic showing the differential E–P interaction (bottom left). Box centerline, median; box limits, upper and lower quartiles; box whiskers, 1.5× interquartile range. Tracks visualize HiChIP 1D H3K27ac enrichment, interaction profiles of the −9 kb enhancer and significant loop interactions colored by adjusted *P* value (bottom right). *P* values were calculated using a two-sided binomial test and corrected using the Benjamini–Hochberg procedure. Two alternative TSS for *ESR1* are annotated; the enhancer is -9kb from the ENST00000440973.5 TSS and looping interactions are analyzed for the ENST00000206249.7 TSS. **h**, H3K27ac HiChIP signal z score across patients for E–P interactions between *ATF7IP*, *PLBD1*, *C12orf60*, *RERG* and *EPS8* promoters and H3K27ac peak at the *H4-16* locus (top of top panel). H3K27ac 1D signal z score across patients for the *H4-16* H3K27ac peak (top of bottom panel). Heatmap of *ATF7IP*, *PLBD1*, *C12orf60*, *RERG* and *EPS8* RNA expression and schematic showing the differential E–P interactions (bottom left). Tracks visualize HiChIP 1D H3K27ac enrichment, interaction profiles of the *H4-16* H3K27ac peak and significant loop interactions colored by adjusted *P* value (bottom right). *P* values were calculated using a two-sided binomial test and corrected using the Benjamini–Hochberg procedure.

Dimensionality reduction of either H3K27ac peak or HiChIP loop signal, followed by *t*-distributed stochastic neighbor embedding, also separated samples by cancer type and was consistent with previously described ATAC–seq clusters (Extended Data Fig. 3b–d)^12^. Additionally, sample clustering reflected additional features, such as separation between basal and nonbasal breast cancers (Extended Data Fig. 3e) and differences between esophageal squamous cell carcinoma (ESCC) and esophageal adenocarcinoma (EAC; Extended Data Fig. 3f)^33^. To identify differential H3K27ac peaks and HiChIP loops, we used feature binarization^12,34^ to identify features that are unique to a specific cancer type or subset of cancer types and identified 28,716 differential H3K27ac peaks and 5,073 differential loops (Extended Data Fig. 4a,b). Consistent with prior results from chromatin accessibility profiling, cancer-type-specific peaks and loops identified by HiChIP were enriched for relevant transcription factor (TF) motifs, including p63 in squamous cancers (ESCC and lung squamous cell carcinoma (LUSC)) and androgen response elements in prostate adenocarcinomas (PRAD; Extended Data Fig. 4c,d). Interestingly, we noted that some TFs were preferentially enriched in H3K27ac-associated loops relative to H3K27ac peaks, suggesting that these TFs may potentially be more relevant for 3D looping interactions. Expanding on our observation of cancer-type-specific regulation of *MYC*, we identified 51 oncogenes with >5 linked differential H3K27ac peaks, nominating tissue-specific regulatory elements (Extended Data Fig. 4e and Supplementary Table 3).

Furthermore, we noted multiple loci that were enriched for H3K27ac in multiple cancer types but engaged in differential looping in specific cancer types, although most differential peaks overlapped with a differential loop (Extended Data Fig. 4f). For example, we identified a putative regulatory element located −9 kb of the *ESR1* gene encoding estrogen receptor α that is marked by H3K27ac in nonbasal breast invasive carcinomas (BRCA), thyroid carcinoma (THCA) and uterine corpus endometrial carcinoma (UCEC), but with increased looping to the *ESR1* promoter in UCEC, which correlates with higher *ESR1* expression (Extended Data Fig. 4g). Additionally, we identified more complex examples, such as an H3K27ac peak overlapping histone H4 gene *H4-16* with differential looping interactions to several nearby genes that correlates with the expression of the interacting gene (Extended Data Fig. 4h). These results suggest that 3D cancer genomes have globally similar compartment organization, but enhancer-associated histone modifications and fine-scale E–P loops distinguish different cancer types.

### Oncogene expression by enhancer rewiring or CN gain

**Extended Data Fig. 5:**
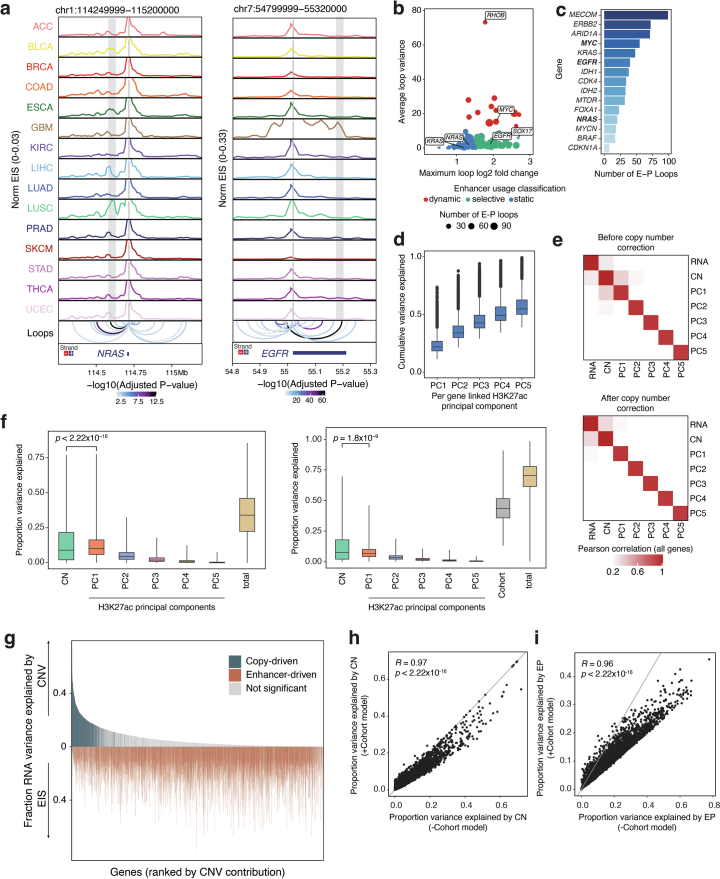
Modeling variance in RNA expression explained by copy number or enhancer activity. **a**, H3K27ac HiChIP interaction profiles for *NRAS* and *EGFR* for all samples merged by cancer type (right). Significant loop interactions colored by adjusted *P* value shown below. *P* values were calculated using a two-sided binomial test and corrected using the Benjamini–Hochberg procedure. **b**, Scatter plot of average loop variance per oncogene between cancer types versus maximum log_2_(fold change) colored by oncogene classification. **c**, Bar plot of unique enhancer–promoter loops for indicated oncogenes. **d**, Box plot of cumulative variance explained by top 5 principal components (PCs) of H3K27ac signal. Each point represents a gene (n = 11,324 genes with linked H3K27ac peaks for each PC). Box centerline, median; box limits, upper and lower quartiles; box whiskers, 1.5× interquartile range. **e**, Heatmap of average Pearson correlation between RNA expression, CNV and top 5 H3K27ac PCs of for all genes (n = 12,570) before and after copy-number regression. **f**, Box plot of variance explained per gene by CNV, top 5 H3K27ac PCs and all variables (left). Box plot of variance explained per gene by CNV, top 5 H3K27ac PCs, cancer type and all variables (right). *P* value determined by two-sided Wilcoxon rank-sum test and not adjusted for multiple comparisons. Box plot components as in (**d**). **g**, All genes with variance in RNA expression >1 (n = 5,985) ranked by fraction of RNA variance explained by CNV across cancer samples, modeled without including cancer type as a variable. Each column is a gene. Genes highlighted on top are significantly (adjusted *P* value < 0.05) explained by CNV (dark blue), while genes highlighted on the bottom are significantly (adjusted *P* value < 0.05) explained by E–P signal (orange). *P* values were calculated using a two-sided t test and corrected using the Benjamini–Hochberg procedure. **h**, Scatter plot of proportion variance explained by copy number with and without including cancer type in regression analysis. *P* value determined by two-sided t test and not adjusted for multiple comparisons. **i**, Scatter plot of proportion variance explained by H3K27ac signal with and without including cancer type in regression analysis. *P* value determined by two-sided t test and not adjusted for multiple comparisons.

We next examined the roles of the 3D genome in oncogene transcription. We focused on 110 consensus driver oncogenes that were found to be recurrently mutated or overexpressed across different cancer types^35^. The 3D chromatin landscape across cancer types suggested the following three classifications of enhancer usage: (1) static enhancer usage, exemplified by *NRAS* (encoding neuroblastoma RAS viral oncogene homolog); (2) selective enhancer connectivity in one cancer type, such as *EGFR* (encoding epidermal growth factor receptor) in glioblastoma; and (3) highly dynamic patterns of enhancer contacts, including *MYC* (encoding MYC proto-oncogene, bHLH transcription factor; Fig. 1d, Extended Data Fig. 5a,b and Supplementary Table 4). Individual oncogenes varied considerably in the number of E–P loops identified by HiChIP, suggesting that enhancer activity may contribute to RNA expression in a gene-specific manner (Extended Data Fig. 5c).

**Fig. 2:**
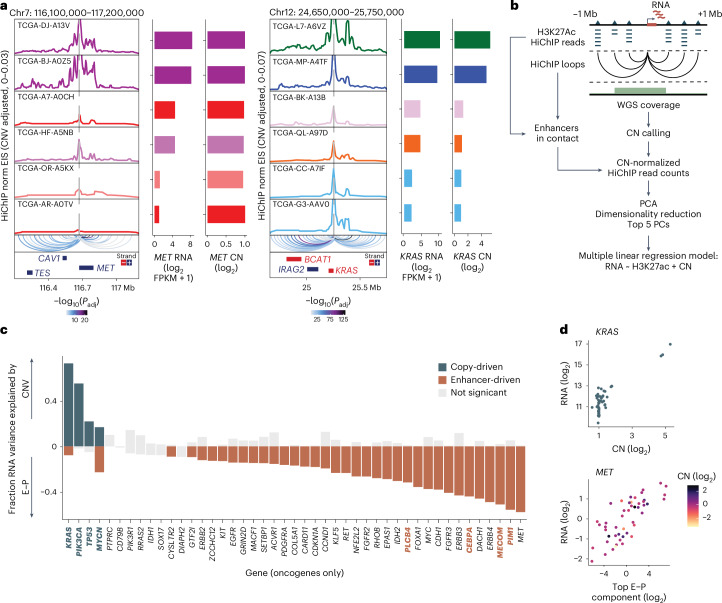
Differential contributions of CN and enhancer activity explain variability in oncogene expression. **a**, Interaction profiles of the *MET* and *KRAS* promoters for individual samples with high (rank 1 and 2 of 56 samples with matched RNA-seq, WGS and HiChIP data), intermediate (rank 28 and 29) or low (rank 55 and 56) RNA expression with significant loop interactions colored by adjusted *P* value. *P* values were calculated using a two-sided binomial test and corrected using the BH procedure. Bar plots visualize RNA expression and CN inferred from WGS. **b**, Schematic representation of analysis to infer contribution of enhancer interaction gain or gene CN to oncogene mRNA expression level. **c**, Oncogenes with variance in RNA expression >1 (*n* = 45) ranked by the fraction of RNA variance explained by CNV or linked enhancer activity across cancer samples. Each column is a gene. Genes with dark blue-colored bars on the top are significantly explained by CNV, while genes with orange-colored bars on the bottom are significantly explained by enhancer signal (E–P; H3K27ac term with the highest relative importance for each gene is shown). Genes in bold dark blue or orange text are also significant when cancer type is included in regression analysis. **d**, Scatter plot of the relationship between DNA CN and RNA expression for copy-driven gene *KRAS* (top) and E–P interaction signal and RNA expression for enhancer-driven gene *MET* (bottom). FPKM, fragments per kilobase of transcript per million mapped reads.

In addition to enhancer rewiring, DNA CN has a profound effect on oncogene expression. Not only do amplified genes tend to be more highly expressed due to additional DNA copies, but they may also explore different gene regulatory space^19,20,36^. We first compared CN and enhancer activity for cases with low, intermediate or high RNA expression and found variable contributions depending on the gene. For example, *MET* showed a strong correlation between H3K27ac HiChIP signal and RNA expression with minimal changes in DNA CN (Fig. 2a). In contrast, differences in *KRAS* RNA expression reflected DNA CNVs while H3K27ac HiChIP signal was largely unchanged. To determine the relative contributions of both enhancer usage and CNVs on oncogene transcription, we performed an integrated analysis using H3K27ac HiChIP, bulk RNA-seq and WGS. We used multiple linear regression to determine the relative contributions of DNA CN and enhancer interaction score to variance in RNA expression across all driver oncogenes and cancer types (Fig. 2b). To account for multiple coordinated enhancers, for each gene, we identified all significant HiChIP looping interactions as well as overlapping H3K27ac peaks and took the top five principal components of H3K27ac signal across all samples (Extended Data Fig. 5d). We noted correlations between DNA CN and the first principal component of H3K27ac signal, which was mitigated by CN regression (Extended Data Fig. 5e).

**Extended Data Fig. 6:**
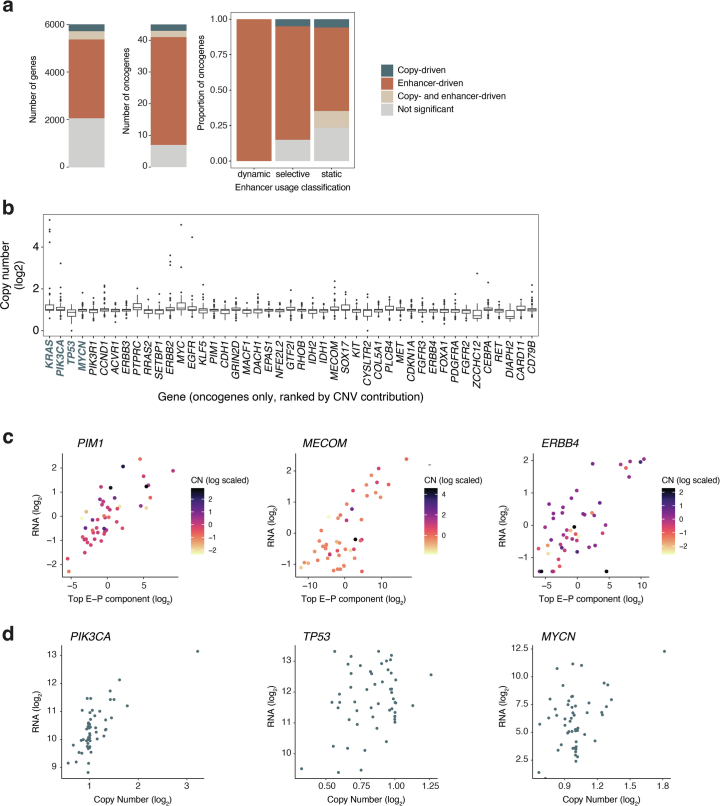
Copy-driven and enhancer-driven gene classification. **a**, Stacked bar plot of gene classification based on whether variance in RNA expression is significantly explained by DNA copy number, enhancer activity, both or neither based on multiple linear regression analysis for all genes (left), oncogenes (middle) or oncogenes grouped by enhancer usage classification (right). Genes with variance in RNA expression >1 included in modeling analysis (n = 5,985 total genes and n = 45 oncogenes). **b**, Box plot of copy-number distribution for all oncogenes, ranked by CNV contribution in regression analysis for all samples included in analysis (n = 62). Box centerline, median; box limits, upper and lower quartiles; box whiskers, 1.5× interquartile range. **c**, Scatter plot of the relationship between top E–P component and RNA expression for enhancer-driven genes *PIM1*, *MECOM* and *ERBB4*. **d**, Scatter plot of the relationship between DNA copy number and RNA expression for copy-driven genes *PIK3CA*, *TP53* and *MYCN*.

Overall, we found that both H3K27ac signal and DNA CN explained variance in RNA expression, although individual genes differed substantially in how much variance in RNA expression could be explained by either CN or enhancer activity (Fig. 2c and Extended Data Fig. 5f,g). Given the prevalence of cancer-type-specific enhancers, we also performed regression analysis with cancer type included and found that while cancer type explains a considerable proportion of variance and reduces the variance explained by E–P signal, the variance explained per gene for both CN and E–P signal is highly correlated in both analyses (Extended Data Fig. 5f,h,i). Quantitative analysis showed that for the majority of all genes and over 70% of oncogenes, mRNA expression is better explained by gains in enhancer activity, while expression of the remaining genes is better explained by DNA CN (Fig. 2c and Extended Data Fig. 6a). When comparing to patterns of static, selective or dynamic enhancer usage as defined above, we find that only oncogenes with selective and static enhancer usage were copy-driven, while all classes of enhancer usage can be enhancer-driven (Extended Data Fig. 6a). While some of the top copy-driven oncogenes have more extreme variation in CN, several enhancer-driven oncogenes have comparable variation in CN, suggesting that gene classification is not solely driven by extreme changes in CN (Extended Data Fig. 6b). The pattern of enhancer or copy-driven oncogene expression is remarkably binary and consistent (Fig. 2d and Extended Data Fig. 6c,d). This analysis demonstrates that CN amplification explains overexpression for a few oncogenes, while enhancer activity better accounts for most cases, highlighting the role of the 3D regulatory landscape in oncogene activation.

### Cell-type-specific E–P loops in the tumor microenvironment (TME)

**Fig. 3:**
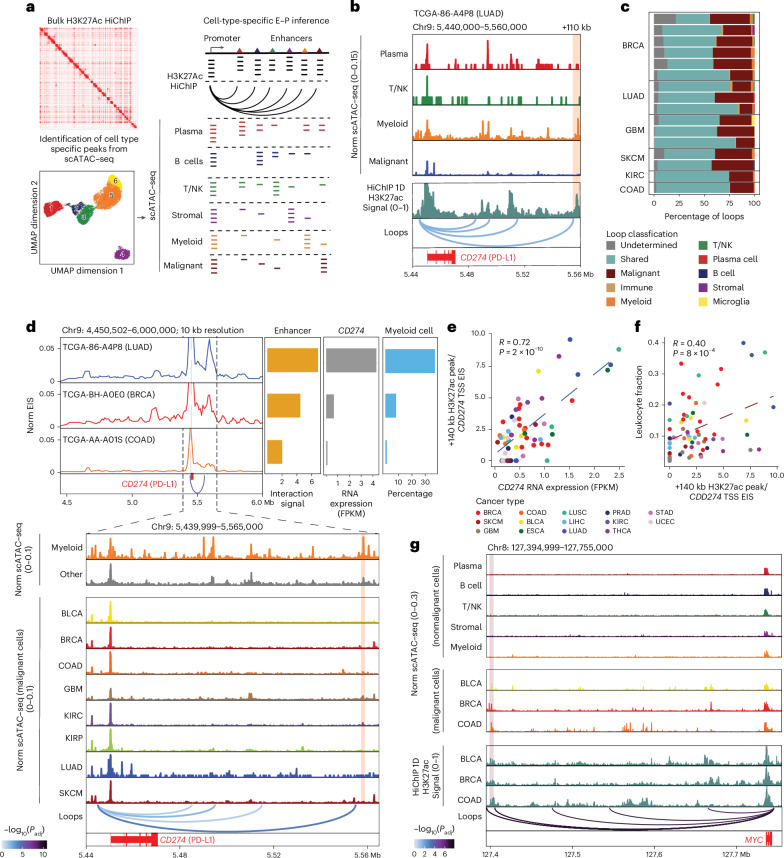
Deconvolution of HiChIP signal resolves malignant and immune cell-specific chromatin conformation in TME. **a**, Schematic representation showing identification of cell-type-specific enhancer–promoter interactions using integration of HiChIP and scATAC–seq data. **b**, Signal tracks showing scATAC–seq and H3K27ac HiChIP at *CD274* locus (encoding PD-L1) for sample TCGA-86-A4P8. The scATAC–seq track indicates the chromatin accessibility of different cells in TME (top). The H3K27ac HiChIP track indicates the bulk H3K27ac signal (middle). The interaction track indicates the *CD274* promoter-associated interactions. The shaded area indicates the myeloid cell-specific H3K27ac peak. **c**, Bar plot of loop annotation based on scATAC–seq/HiChIP integration for samples with matched scATAC and H3K27ac HiChIP. **d**, Integrative virtual 4C and scATAC–seq signal tracks showing the myeloid cell-specific enhancer–promoter interaction for *CD274* (encoding PD-L1). The virtual 4C plot shows the EIS changes (left) with matched *CD274* RNA expression and myeloid cell percentages based on scATAC–seq (right). The scATAC–seq track indicates the chromatin accessibility of myeloid cells, noncancer cells and cancer cells across eight different cancer types (bottom). The marked area indicated the myeloid cell-specific H3K27ac peak. Significant loop interactions are colored by adjusted *P* value, and *P* values were calculated using a two-sided binomial test and corrected using the BH procedure. **e**, Scatter plot showing the correlation between the enhancer–promoter interaction and *CD274* RNA expression. The correlation coefficient was calculated using Pearson correlation, and the *P* value was calculated using a two-sided *t* test. **f**, Scatter plot showing the correlation between the enhancer–promoter interaction and RNA-seq-derived leukocyte fraction estimation. The correlation coefficient was calculated using Pearson correlation, and the *P* value was calculated using a two-sided *t* test. **g**, Signal tracks showing the integrative track of scATAC–seq and H3K27ac HiChIP at *MYC* locus. The scATAC–seq track indicates the chromatin accessibility of different noncancer and cancer cells in eight cancer types (top). The H3K27ac HiChIP track indicates the bulk level H3K27ac signal in BLCA, BRCA and COAD (middle). The interaction track indicates the *MYC* promoter-associated interactions. The shaded area indicates H3K27ac peaks that overlap with cancer risk-associated SNPs. Significant loop interactions are colored by adjusted *P* value, and *P* values were calculated using a two-sided binomial test and corrected using the BH procedure.

**Extended Data Fig. 7:**
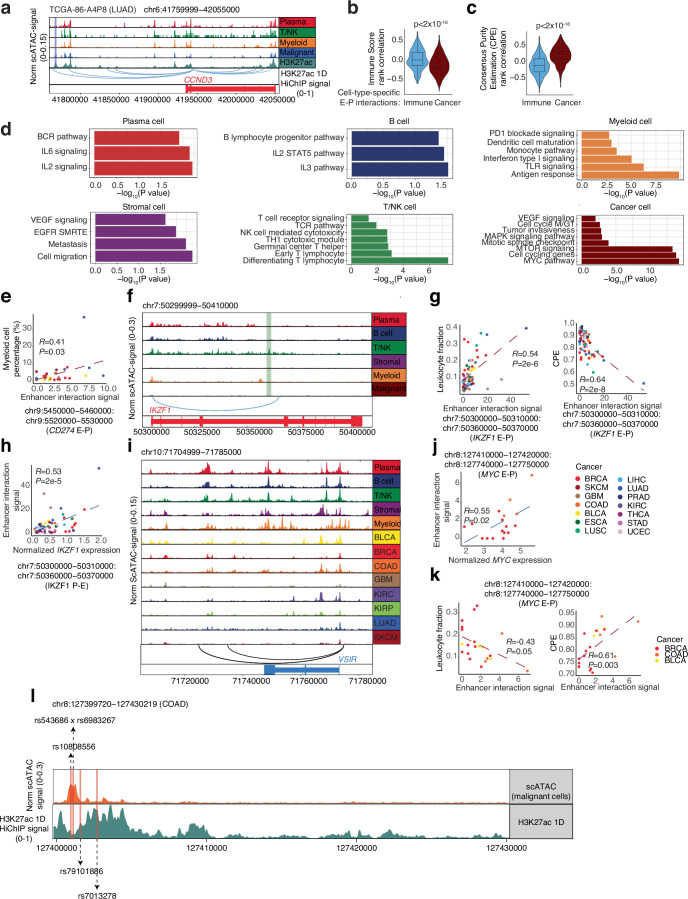
Validation of HiChIP deconvolution framework in tumor microenvironment. **a**, Signal tracks at the *CCND3* locus. scATAC–seq track shows chromatin accessibility in TCGA-86-A4P8 cells (top), H3K27ac HiChIP track shows bulk H3K27ac signal (middle) and interaction track indicates promoter-associated loops. Shaded region marks a cancer-cell-specific H3K27ac peak. **b**, Violin and box plot showing differences in ImmuneScore correlation coefficients between immune cell-specific (n = 1,029) and cancer-cell-specific (n = 1,551) enhancer–promoter (E–P) interactions. *P* value was calculated using a two-sided Wilcoxon rank-sum test. **c**, Violin and box plot comparing correlation with tumor purity (CPE score) between immune- and cancer-cell-specific E–P interactions. *P* value calculated using a two-sided Wilcoxon rank-sum test. In (**b**,**c**), box centerline denotes median; box limits, upper and lower quartiles; whiskers, 1.5× interquartile range. **d**, Bar plot showing Gene Ontology enrichment of genes regulated by cell-type-specific E–P interactions. *P* values were determined using two-sided Fisher’s exact test. **e**, Scatter plot showing correlation between E–P interaction strength and myeloid cell fraction. Correlation coefficient calculated using Pearson correlation; *P* value by two-sided t test. **f**, Signal tracks at the *IKZF1* locus showing merged scATAC–seq signal across eight cancer types (top) and H3K27ac HiChIP interactions (bottom). Shaded region indicates a T/NK cell-specific H3K27ac peak. **g**, Scatter plots showing correlation between *IKZF1* E–P interaction and leukocyte fraction (left) or CPE tumor purity score (right), with Pearson correlation coefficients and *P* values from two-sided t-tests. **h**, Scatter plot showing correlation between *IKZF1* E–P interaction and *IKZF1* RNA expression. Correlation was calculated using Pearson correlation; *P* value by a two-sided t test. **i**, Signal tracks at the *VSIR* locus showing scATAC–seq signal in noncancer and cancer cells across eight cancer types (top) and promoter-associated interactions (bottom). **j**, Scatter plot showing correlation between *MYC* E–P interaction and *MYC* RNA expression, with Pearson correlation coefficient and *P* value from two-sided t test. **k**, Scatter plots showing correlation between *MYC* E–P interaction and leukocyte fraction (left) or CPE tumor purity score (right), with Pearson correlation coefficients and two-sided t test *P* values. **l**, Signal tracks showing scATAC–seq and H3K27ac HiChIP signal at a *MYC* enhancer in COAD, with shaded regions indicating known COAD risk-associated SNPs.

Epigenetic regulation of immune cells profoundly impacts cancer development; however, knowledge regarding enhancer–promoter interactions in the TME is limited. We developed a computational framework to deconvolute H3K27ac HiChIP into cell-type-specific signals using patient-matched single-cell ATAC–seq (scATAC–seq)^37^ (Fig. 3a and Supplementary Table 5; Methods). For instance, we identified a myeloid cell-specific enhancer–promoter interaction for the *CD274* gene (encoding programmed death-ligand 1 (PD-L1)) in lung adenocarcinoma (LUAD) sample TCGA-86-A4P8 (Fig. 3b). HiChIP revealed an interaction between the *CD274* promoter and a regulatory element marked by H3K27ac located +110 kb away, adjacent to previously described enhancers^38^. scATAC–seq analysis from the same sample validated myeloid-specific accessibility at this enhancer, with minimal accessibility in malignant or other immune cells. In contrast, an enhancer −140 kb away from the promoter of the *CCND3* gene (cyclin D3) displayed chromatin accessibility specific to malignant cells (Extended Data Fig. 7a).

We extended this framework to 29 patients with matched H3K27ac HiChIP and scATAC–seq, focusing on 16 samples with sufficient nonmalignant cells for scATAC–seq peak calling (Methods). Most E–P interactions overlapped with scATAC–seq peaks that were accessible across multiple cell types; however, we were able to identify cell-type-specific interactions (Fig. 3c). In total, we identified 1,551 malignant cell-specific and 745 immune cell-specific interactions. Immune cell-associated E–P interactions displayed significantly lower correlation with tumor purity and higher correlation with RNA-seq-derived leukocyte fraction estimates compared to malignant cell-associated E–P interactions (Extended Data Fig. 7b,c; Methods)^39,40^. Gene Ontology analysis revealed that malignant cell enhancer contacts were enriched for cell division and growth genes, while those in tumor-associated myeloid, B and T/natural killer (NK) cells were linked to immune pathways (Extended Data Fig. 7d).

PD-L1, encoded by *CD274*, is a ‘don’t kill me’ signal that dampens anticancer T cell responses and is a major target for cancer immunotherapy^41^. While commonly expressed by malignant cells, PD-L1 is also highly expressed by immune cells in the TME, including macrophages and dendritic cells^42^. We identified a dynamic enhancer located 110 kb 3′ of *CD274* with E–P interaction signal correlated with *CD274* mRNA expression, leukocyte fraction estimation and myeloid cell frequency estimated by scATAC–seq (Fig. 3d–f, Extended Data Fig. 7e and Supplementary Table 6; Methods). Pseudobulk single-cell chromatin accessibility analysis further supported the myeloid specificity of this enhancer, which was uniquely accessible in myeloid cells (Fig. 3d). We also examined T/NK cell-specific E–P interactions for *IKZF1*, a known regulator of immune cell development expressed by multiple immune cell types, including T cells^43^. While the *IKZF1* promoter is accessible across multiple immune cell types in the TME, we identified an intronic, T/NK cell-specific enhancer with significant looping to the promoter (Extended Data Fig. 7f). The *IKZF1* E–P interaction signal correlated positively with *IKZF1* RNA expression as well as leukocyte fraction estimation but negatively with tumor purity estimation (Extended Data Fig. 7g,h). In addition, many E–P interactions exhibited shared chromatin accessibility between malignant and immune cells, including immune checkpoint genes like *CTLA4*, *TIGIT*, *VSIR* and *TIM3* (refs. ^44,45^; Supplementary Table 6 and Extended Data Fig. 7i). These results suggest that the immunological setpoints of cancers reflect the contributions of multiple cell types in the TME.

scATAC–seq-based deconvolution enabled the classification of malignant cell-specific E–P interactions, nominating enhancers linked to altered gene expression in transformed cells (Fig. 3c). Gene Ontology analysis revealed that one of the most significantly enriched sets of enhancer target genes is the MYC pathway (Extended Data Fig. 7d). We enumerated malignant cell-specific E–P loops at the *MYC* locus in BLCA, BRCA and COAD samples (Fig. 3g). *MYC* EIS positively correlated with *MYC* mRNA expression and tumor purity estimation but negatively correlated with leukocyte fraction estimation (Extended Data Fig. 7j,k). Genome-wide association studies have identified numerous noncoding variants associated with increased risk of cancer. Seven SNPs associated with cancer risk map to the cancer-specific *MYC* enhancers (Extended Data Fig. 7l), including the COAD risk variant rs6983267 that has been replicated in multiple cohorts^46–50^, suggesting that these variants exert their effect by impacting MYC expression in transformed cells rather than immune or stromal cells. We extend this SNP analysis to all malignant cell-specific E–P interactions, providing a comprehensive list of risk SNPs linked to target genes (Supplementary Table 7).

### Three-dimensional genome reveals targets of noncoding regulatory mutations

**Fig. 4:**
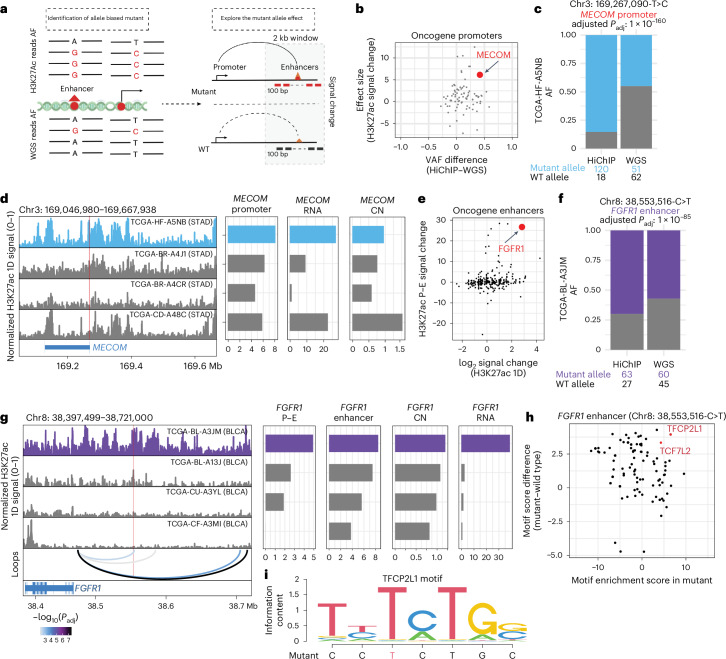
Integration of WGS and HiChIP identifies cancer-relevant regulatory mutations and target genes. **a**, Schematic representation showing the workflow of identifying the H3K27ac-associated noncoding mutations. **b**, Scatter plot indicating the relationship between oncogene promoter-associated HiChIP and WGS allele frequency differences and the effect size (*T* score) of the associated H3K27ac signal change between mutant and wild-type patients. The *T* score was calculated by a two-sided *t* test. **c**, Bar plot showing the allele frequency of chr3: 169,267,090-T>C (*MECOM*) mutant between HiChIP and WGS for sample TCGA-HF-A5NB (STAD). The *P* value was calculated by Fisher’s exact test and corrected using the BH procedure. **d**, Signal tracks showing the integrative track of H3K27ac HiChIP at *MECOM* locus normalized by reads in TSS. The H3K27ac 1D signal track indicates the bulk level H3K27ac signal in STAD samples (left). Mutant patient TCGA-HF-A5NB is highlighted in blue. The chr3: 169,267,090-T>C mutant position is labeled in red line. Bar plots indicate matched H3K27ac signal (CN corrected), *MECOM* expression and CN at *MECOM* locus. **e**, Scatter plot quantifying the relationship between enhancer activity and enhancer–promoter interaction changes for oncogene-associated enhancers with somatic variants. **f**, Bar plot showing the allele frequency of chr8: 38,553,516-C>T (*FGFR1* enhancer) mutant between HiChIP and WGS for sample TCGA-BL-A3JM (BLCA). The *P* value was calculated by Fisher’s exact test and corrected using the BH procedure. **g**, Signal tracks showing the integrative track of HiChIP 1D H3K27ac enrichment at *FGFR1* locus normalized by reads in TSS. The H3K27ac 1D signal track indicates the bulk level H3K27ac signal (CN corrected) and *FGFR1* enhancer–promoter interactions in BLCA samples (left). Mutant patient TCGA-BL-A3JM is highlighted in purple. The chr8: 38,553,516-C>T mutant position was labeled in red line. Bar plots indicate matched H3K27ac signal, *FGFR1* expression and CN at *FGFR1* locus. Significant loop interactions are colored by adjusted *P* value, and *P* values were calculated using a two-sided binomial test and corrected using the BH procedure. **h**, Scatter plot indicating the association between chr8: 38,553,516-C>T mutant-involved motif enrichment changes and motif enrichment scores in chr8: 38,553,516-C>T mutant region. **i**, Motif sequence plot showing the overlap between the mutant sequence and the enriched motif sequence for TFCP2L1. AF, allele frequency.

**Extended Data Fig. 8:**
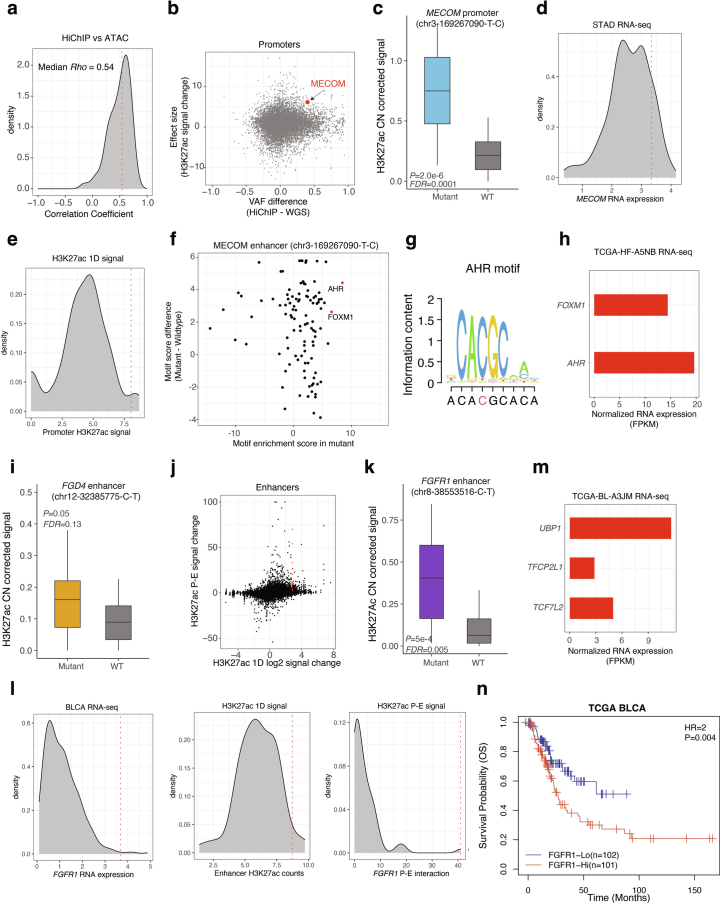
Validation of noncoding mutation-associated H3K27ac signal change. **a**, Density plot showing distribution of correlation coefficients between mutant allele frequencies derived from H3K27ac HiChIP and ATAC data. **b**, Dot plot showing relationship between promoter-associated HiChIP and WGS allele frequency differences and effect size (*T* score) of corresponding H3K27ac signal changes between mutant and wild-type patients. *T* score was calculated using a two-sided t test. **c**, Box plot showing H3K27ac signal differences in the chr3:169267090-T>C region (±1 kb) between mutant (n = 20 bins from one sample) and wild-type patients (n = 60 bins from three samples). *P* value calculated by two-sided t test and adjusted using Benjamini–Hochberg procedure. Box centerline, median; box limits, upper and lower quartiles; whiskers, 1.5× interquartile range. **d**, Density plot showing distribution of *MECOM* expression in stomach cancer RNA-seq cohort; mutant patient labeled by a red dashed line. **e**, Density plot showing distribution of H3K27ac signal at the *MECOM* promoter in the TCGA HiChIP cohort; mutant patient labeled in red dashed line. **f**, Dot plot showing association between mutant-involved motif enrichment changes at chr3:169267090-T>C and motif enrichment scores. **g**, Motif sequence plot showing overlap between the mutated sequence and the enriched AHR motif. **h**, Bar plot showing RNA expression of enriched transcription factors *FOXM1* and *AHR* in the TCGA-HF-A5NB RNA-seq dataset. **i**, Box plot showing H3K27ac signal difference in the chr12: 32385775-C>T region (±1 kb) between mutant and wild-type patients. *P* value calculated by two-sided t test and corrected using Benjamini–Hochberg method. **j**, Volcano plot showing association between enhancer mutations and changes in enhancer activity and enhancer–promoter interactions. **k**, Box plot showing H3K27ac signal difference in the chr8: 38553516-C>T region (±1 kb) between mutant and wild-type patients. Statistical testing as in (**i**). **l**, Density plots showing distribution of *FGFR1* expression, enhancer H3K27ac signal, and enhancer–promoter interactions in the TCGA cohort, with mutant patient values marked by red dashed lines. **m**, Bar plot showing expression of enriched transcription factors *UBP1*, *TFCP2L1* and *TCF7L2* in TCGA-BL-A3JM RNA-seq data. **n**, Kaplan–Meier plot showing prognostic value of *FGFR1* expression; patients stratified into high and low groups based on top and bottom 25% percentiles. *P* value by log-rank test.

Identification of somatic mutations in active regulatory elements with higher allele frequencies in H3K27ac HiChIP compared to WGS can nominate noncoding mutations that may promote enhancer activity to drive cancer initiation and progression (Fig. 4a). Building on prior efforts using WGS as well as ATAC–seq to nominate functional noncoding variants^12,51^, additional WGS and HiChIP data generated in this study provide additional power to nominate functional variants and to identify target genes. Using somatic mutations identified by WGS, we calculated mutant allele frequencies in H3K27ac HiChIP, achieving a median correlation of 0.54 with ATAC–seq data (Extended Data Fig. 8a). We then quantified the mutant allele’s impact on enhancer activity based on the average H3K27ac signal changes within a 2-kb region centered on the single-nucleotide variant relative to all cases with only the reference allele (Fig. 4a; Methods). We identified 7,517 somatic mutations (2,975 promoter mutations and 4,542 enhancer mutations) with higher variant allele frequency in H3K27ac HiChIP over WGS (Fig. 4a and Extended Data Fig. 8b; Methods), suggesting enhanced regulatory activity.

Among oncogene promoter variants, this analysis nominated a stomach cancer-associated variant (chr3: 169,267,090-T>C) in the *MECOM* promoter, showing a higher allele frequency in HiChIP (85%) than WGS (45%; Fig. 4b,c) and increased H3K27ac signal (Extended Data Fig. 8c). Furthermore, a concordant trend between H3K27ac signal changes and mRNA expression levels was observed across different patients, except for sample TCGA-CD-A48C, which had high RNA expression despite modest H3K27ac signal at the *MECOM* promoter. Examination of WGS data revealed a focal amplification of the *MECOM* locus for this sample, suggesting that either noncoding promoter mutation or gene copy amplification can promote oncogene overexpression (Fig. 4d). Indeed, *MECOM* RNA expression and H3K27ac promoter signal for the sample with the chr3: 169,267,090-T>C variant rank in the top 16% of TCGA STAD RNA-seq and top 5% of pan-cancer H3K27ac HiChIP (Extended Data Fig. 8d,e). As noncoding mutations can create new binding sites for TFs that may promote gene overexpression, we compared motif enrichment scores between *MECOM* chr3: 169,267,090-T>C mutant and wild-type sequences (Extended Data Fig. 8f). Differential motif analysis nominated AHR and FOXM1 as the most significant TF motif gained by the T>C change in the *MECOM* promoter (Extended Data Fig. 8g), and RNA-seq data analysis confirmed the expression of *AHR* and *FOXM1* in the tumor sample (Extended Data Fig. 8h).

We next investigated the presence of enhancer mutations that may impact gene expression and regulatory element activity. We first validated the previously identified *FDG4* enhancer mutation in the BLCA cohort using HiChIP (Extended Data Fig. 8i)^12^. Consistent with ATAC–seq data, the sample with the chr12: 32,385,775-C>T variant showed substantially higher H3K27ac signal compared to noncarriers (Extended Data Fig. 8i). To further nominate functional noncoding variants, we examined both 1D H3K27ac enrichment and E–P looping assessed by HiChIP and nominated 2,214 variants with increased E–P interaction signal (Extended Data Fig. 8j). The chr8: 38,553,516-C>T variant linked to the *FGFR1* promoter in BLCA exhibited allelic bias in HiChIP data and an eightfold increase in H3K27ac signal (Fig. 4e–g and Extended Data Fig. 8k). This variant dramatically enhanced E–P interaction signal (1.4- to 70-fold) and *FGFR1* expression, ranking in the top 1% of the BLCA cohort, without evidence of CNVs (Fig. 4g and Extended Data Fig. 8l). Differential motif analysis revealed that the C to T change created a new binding motif for the TFCP2L1 TF (Fig. 4h,i), which is associated with cell cycle progression and stemness during bladder cancer progression^52^ and is highly expressed in the affected sample (Extended Data Fig. 8m). Finally, high *FGFR1* expression correlated with worse prognosis in BLCA, suggesting functional consequences of this enhancer-associated noncoding mutation (Extended Data Fig. 8n).

### Extensive enhancer rewiring from structural rearrangements

**Fig. 5:**
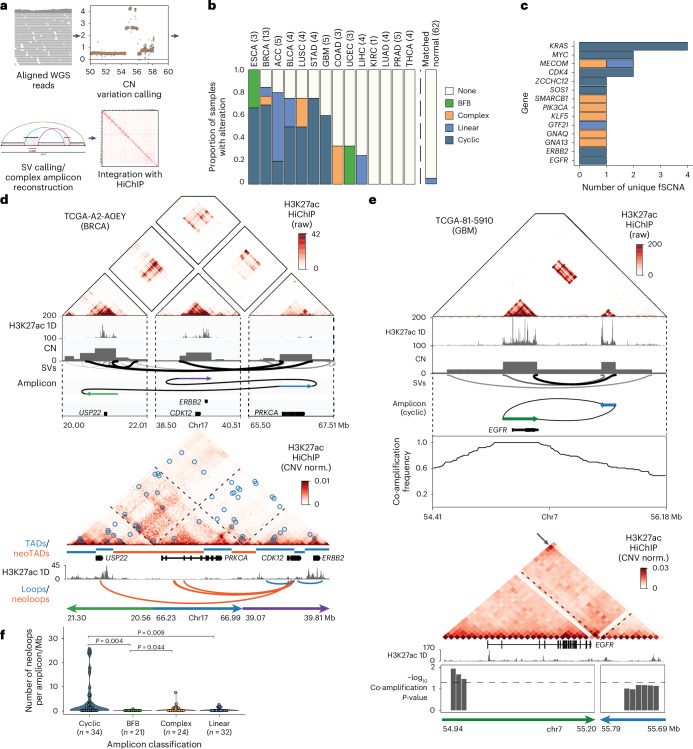
Impact of structural rearrangement and ecDNA amplification on enhancer connectivity. **a**, Workflow of the joint HiChIP–WGS analysis for simple structural variants and complex focal amplifications. **b**, Distribution of cyclic, BFB, complex and linear somatic focal amplifications detected across 62 tumor whole-genome samples with corresponding HiChIP data and 62 patient-matched normal samples as controls. **c**, Distribution of cyclic, BFB, complex, linear fSCNA affecting oncogenes. **d**, Raw HiChIP contact matrix of *ERBB2* rearrangement with tracks visualizing H3K27ac 1D signal enrichment, CN inferred from WGS, SVs identified by WGS and amplicon prediction (top). The raw, unnormalized HiChIP contact matrix allows for visualization of regions of high HiChIP signal before normalization, which correspond to amplifications and structural rearrangements detected by WGS. CN-normalized HiChIP contact matrix with tracks visualizing TADs/neoTADs, H3K27ac 1D signal enrichment and loops/neoloops (bottom). **e**, Raw HiChIP contact matrix of a cyclic (ecDNA-like) *EGFR* rearrangement with tracks visualizing H3K27ac 1D signal enrichment, CN inferred from WGS, SVs identified by WGS, amplicon prediction and co-amplification frequency across all TCGA WGS samples (top). Tracks visualizing H3K27ac 1D signal enrichment and significance of co-amplification with CN-normalized HiChIP matrix below (bottom). Arrow indicates increased interaction signal indicative of a circular amplicon. **f**, Violin and box plot quantifying neoloops per megabase within cyclic, BFB, complex, linear amplifications identified by NeoLoopFinder (*n* = number of unique amplifications). Loop counts are quantified for each focal amplification, normalized by the size of the focal amplification and classified as a neoloop if they span an SV breakpoint. *P* values were calculated using a two-sided Wilcoxon rank-sum test and adjusted using the BH procedure. Box centerline, median; box limits, upper and lower quartiles; box whiskers, 1.5× interquartile range. fSCNA, focal somatic CN amplifications.

**Extended Data Fig. 9:**
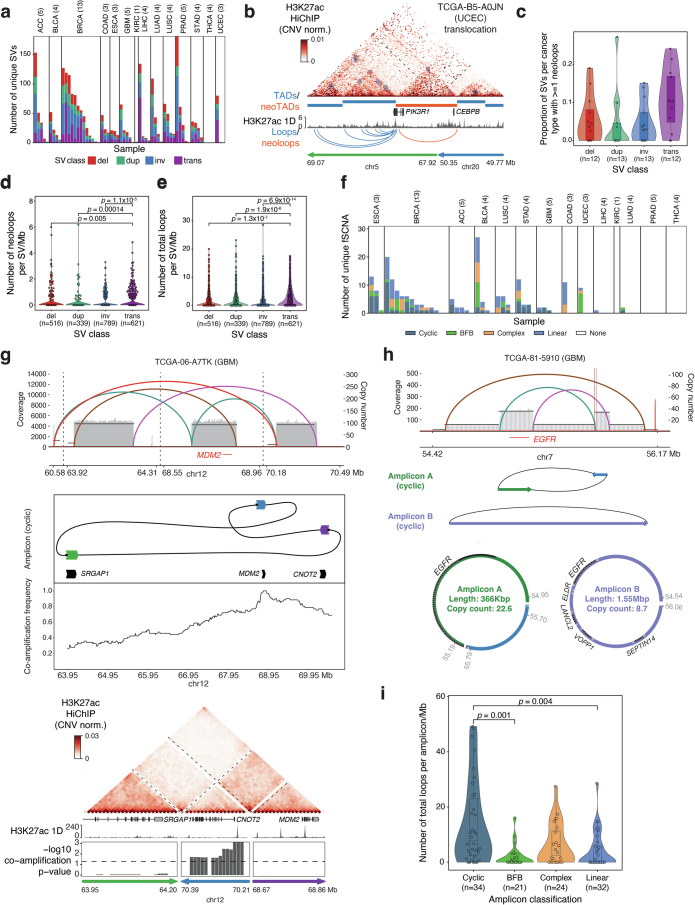
Structural rearrangements affecting enhancer rewiring. **a**, Distribution of simple SVs detected across individual samples (del = deletion, dup = duplication, inv = inversion, trans = translocation). **b**, Copy-number-normalized HiChIP contact matrix for *PIK3R1* translocation with tracks visualizing TADs/neoTADs, H3K27ac 1D signal enrichment and loops/neoloops. **c**, Box and violin plots of the proportion of SVs per cancer type with ≥1 neoloop detected (n = number of cancer types). SVs that overlap with focal amplification breakpoints identified by AmpliconArchitect are excluded in **c**–**e**. Box centerline, median; box limits, upper and lower quartiles; box whiskers, 1.5× interquartile range. **d**, Box and violin plots of the number of neoloops per SV per megabase (n = number of SVs). Box centerline, median; box limits, upper and lower quartiles; box whiskers, 1.5× interquartile range. **e**, Box and violin plots of the number of total loops per SV per megabase (n = number of SVs). Box centerline, median; box limits, upper and lower quartiles; box whiskers, 1.5× interquartile range. **f**, Distribution of cyclic, BFB, complex, linear focal somatic copy-number amplifications (fSCNA) detected across individual samples. **g**, Cyclic structural rearrangement predicted by AmpliconArchitect affecting the *MDM2* locus (top). Amplicon structure and co-amplification frequency across all TCGA WGS samples (middle). Tracks visualizing H3K27ac 1D signal enrichment and significance of co-amplification with copy-number normalized HiChIP matrix below (bottom). **h**, Cyclic structural rearrangement predicted by AmpliconArchitect affecting the *EGFR* locus (top). Schematic of predicted ecDNA structures (bottom). **i**, Number of loops within cyclic, BFB, complex, linear amplifications identified by NeoLoopFinder. Loop counts are quantified for each focal amplification, normalized by the size of the focal amplification. *P* values were calculated using a two-sided Wilcoxon rank-sum test and adjusted using the Benjamini–Hochberg procedure. Box centerline, median; box limits, upper and lower quartiles; box whiskers, 1.5× interquartile range.

An additional source of somatic alterations with substantial impact on 3D genome organization are structural rearrangements^19,53^. Integration of WGS analysis with H3K27ac HiChIP provides unique insight into the regulatory impact of both simple and complex structural rearrangement events, in particular focal amplifications that can promote oncogene overexpression (Fig. 5a). We first examined the regulatory impact of simple SVs identified by WGS, including deletions, duplications, inversion and translocations (Extended Data Fig. 9a). Rearranging the connectivity of DNA segments can result in both increased contact probability between two previously distant DNA segments and the formation of new TADs and new E–P loops across SV junctions. We used NeoLoopFinder to reconstruct the HiChIP interaction matrices for SVs identified by WGS, such as a translocation linking enhancers on chromosome 20 with the *PIK3R1* oncogene on chromosome 5, and identified new TADs (neoTADs) and new E–P contacts (neoloops), validating the SV reconstruction and nominating new regulatory interactions^54^ (Methods; Extended Data Fig. 9b). Among all classes of simple SVs, we find that translocations tend to have higher proportion of SVs with at least one neoloop and substantially more neoloops/Mb detected per SV as well as more total loops (Extended Data Fig. 9c–e), suggesting that translocations may promote more extensive enhancer rewiring compared to other simple SV classes.

Complex rearrangements link specific amplification classes to distinct DNA repair mechanisms and regulatory features, including breakage-fusion-bridge (BFB) or translocation-bridge^55^ cycles of chromosomal instability and ecDNA formation. Notably, ecDNA amplification, associated with poor clinical outcomes, drives gene overexpression through increased DNA accessibility, enhancer co-amplification and nuclear colocalization^56–59^. Focal genomic amplifications were detected from WGS data using AmpliconArchitect and classified based on the predicted connectivity of discordant breakpoints as linear, complex, cyclic (with head-to-tail connectivity characteristic of ecDNA) or BFB (Fig. 5a,b)^59–61^. Cyclic amplifications associated with ecDNA were one of the most frequent SVs among solid tumors affecting multiple oncogenes, and many tumors exhibit multiple distinct molecular species of ecDNAs (Fig. 5c and Extended Data Fig. 9f).

HiChIP data confirmed the spatial proximity of the three distal genomic segments encompassing the *ERBB2* and *CDK12* genes involved in a complex rearrangement and nominated several new E–P interactions linked to the *CDK12* gene (Fig. 5d). Predicted cyclic amplicons, such as those involving *EGFR* and *MDM2*, were further validated by increased HiChIP interaction frequency at the corner of the matrix (Fig. 5e and Extended Data Fig. 9g). Finally, regulatory elements marked by H3K27ac involved in cyclic amplicons were substantially co-amplified across the TCGA cohort based on WGS data (Fig. 5e and Extended Data Fig. 9g). In addition, we find that ecDNAs exhibit extensive sequence heterogeneity even within individual tumors. In cases where multiple amplicons were nominated by WGS, including multiple cyclic cycles involving *EGFR*, HiChIP provided orthogonal support for the dominating rearrangement, which was supported by a high interaction frequency (Fig. 5e and Extended Data Fig. 9h).

Overall, we find that different classes of rearrangements impact gene regulation at distinct scales, with ecDNA generating the largest number of new E–P loops, as well as larger overall numbers of E–P loops, compared to BFB or linear amplicons (Fig. 5f and Extended Data Fig. 9i). These findings underscore diverse mechanisms of structural rearrangements driving epigenetic rewiring in cancer.

## Discussion

Here we provide an initial survey of 3D genome architecture and enhancer landscape in 15 primary human cancer types. This dataset defined chromosome topology at multiple scales and expanded the lexicon and syntax of gene regulation in cancer. Overlaying 3D genome conformation with DNA mutation, CN, single-cell chromatin accessibility and RNA expression informed how alterations in gene regulation may impact cancer. Nonetheless, due to the range of sequencing depth across archival samples, care should be taken for any pairwise comparison of 3D cancer genomes.

The genome architecture across cancer types is largely conserved in compartments and TADs but varies substantially in E–P loops. This aligns with studies across species and development, suggesting that compartments and TADs serve as stable scaffolds within which dynamic E–P loops regulate gene expression^3,23^. Focusing on driver oncogenes, we observed that CN gain and/or enhancer recruitment can lead to increased RNA expression in a gene-specific manner. Enhancer activity and rewiring better explain mRNA overexpression for most oncogenes, but for a subset, such as *KRAS*, CN gain is the dominant mechanism of overexpression. These findings may guide the clinical profiling of CNVs and regulatory element activity to identify high-risk patients and targeted therapy candidates. We identified noncoding point mutations that can create TF binding sites de novo, leading to enhancer acquisition to activate oncogenes in an allele-specific manner. Although enhancer mutations in cancer are often not recurrent across patients, they can still exhibit potent gene regulatory consequences, and the identification of functional somatic variants affecting oncogenic drivers may enable precision medicine efforts in the future.

The TME comprises a rich ecosystem of malignant and additional cell types. The integration of 3D genome data with single-cell chromatin accessibility nominated cell-type-specific E–P contacts in the TME. We found a major myeloid contribution to immune checkpoint expression, such as PD-L1, consistent with the importance of immunomodulatory tumor-associated macrophages^62^. In contrast, malignant cell-specific E–P loops intersected with SNPs that comprise the major heritable risk alleles for cancer predisposition, supporting the role of cell-autonomous mechanisms for these risk alleles.

SVs drive gene regulatory innovation in cancer by forming new E–P contacts, notably through ecDNA amplification. Unlike chromosomal SVs constrained by TADs, ecDNAs are mobile and unrestricted, driving epigenetic dysregulation and oncogene overexpression in tumor evolution^56,58,59,63,64^. Our analysis of ecDNA amplifications in TCGA samples suggests that subclonal structural rearrangements further enhance ecDNA complexity, generating new E–P loops. This aligns with findings that ecDNAs undergo enhanced mutagenesis and accelerated evolution^65^ and form transcriptionally active hubs that facilitate intermolecular interactions^58,66^, potentially promoting recombination upon DNA damage. As the recognition of mutated oncogenes led ultimately to targeted therapies, understanding 3D genome architecture and gene regulatory circuits may pave the way for new therapeutic strategies in the future.

## Methods

### Ethical approval

This study complied with all relevant ethical regulations and ethical guidance was overseen by the TCGA Program Office. Each study site that contributed biological material had its own ethics board approval. TCGA ethics policies are available at https://www.cancer.gov/ccg/research/genome-sequencing/tcga/history/ethics-policies.

### Tumor sample selection

Samples were selected from the set of samples previously profiled by bulk ATAC–seq^12^ to span the 15 cancer types profiled in this manuscript, with a focus on breast cancer and at least three samples for each other cancer. Within breast cancer, three samples were selected from each major breast cancer subtype (Basal, HER2, LumA and LumB).

### Statistics and reproducibility

Samples were prioritized for selection based on high data quality in previous bulk ATAC–seq experiments, the availability of sufficient nuclei in cryopreserved stocks and the representation of the diversity of cancer types profiled by TCGA. No statistical method was used to predetermine sample size. No data were excluded from the analyses. The experiments were not randomized. The investigators were not blinded to allocation during experiments and outcome assessment. Data distribution was assumed to be normal, but this was not formally tested.

### HiChIP library generation

HiChIP library generation was performed following published protocols^22^. Nuclei used for HiChIP were isolated as part of a previous study^12^ and cryopreserved in BAM Banker. One million cryopreserved nuclei were used per experiment. Briefly, enzyme MboI was used for restriction digestion. Sonication was performed on a Covaris E220 instrument using the following settings: duty cycle 5, peak incident power 140, cycles per burst 200 and time 4 min. All HiChIP was performed using H3K27ac as the target (Abcam, ab4729). Libraries were sequenced on an Illumina HiSeq 4000 with paired-end 75 bp reads. Full protocol details are described in Supplementary Methods.

### Preparation of WGS libraries for cluster amplification and sequencing

In total, 268 TCGA tumor samples were profiled by deep WGS sequencing in this study, and for 263 matched normal samples, WGS was also generated for identification of somatic variants, either collected from peripheral blood (*n* = 255) or from adjacent normal tissue (*n* = 8). Five tumor samples profiled by WGS in this study had previously generated WGS data from normal blood or tissue, which was used in somatic variant identification (Supplementary Table 2). An aliquot of genomic DNA (350 ng in 50 μl) is used as the input into DNA fragmentation (also known as shearing). Shearing is performed acoustically using a Covaris focused-ultrasonicator, targeting 385-bp fragments. Following fragmentation, additional size selection is performed using a solid-phase reversible immobilization cleanup. Library preparation is performed using a commercially available kit provided by KAPA Biosystems (KAPA Hyper Prep without amplification module, KK8505) and with palindromic forked adapters with unique eight-base index sequences embedded within the adapter (purchased from Roche, KK8727). Libraries were sequenced on an Illumina NovaSeq 6000 with paired-end 151-bp reads.

### HiChIP data analysis

HiChIP data were processed as described previously^22^. In brief, paired-end reads were aligned to the hg38 genome using the HiC-Pro pipeline (v.2.11.0)^67^. Default settings were used to remove duplicate reads, assign reads to MboI restriction fragments, filter for valid interactions and generate binned interaction matrices. FitHiChIP (v.8.0) was used to identify loops^68^. Dangling end, self-circularized and religation read pairs were merged with valid read pairs to create a 1D H3K37ac signal bed file, corresponding to H3K27ac ChIP followed by sequencing (ChIP–seq)-like signal that was used for peak calling and 1D signal quantification using standard ChIP–seq analysis tools, including MACS2. FitHiChIP was used to identify ‘peak-to-all’ interactions at 10-kb resolution using peaks called from the 1D HiChIP data using MACS2 (ref. ^69^). Loop calling was restricted to loops with anchors on the same chromosome and separated by 40 kb to 2 Mb. Bias correction was performed using coverage-specific bias. HiChIP loop calling was performed at 10-kb resolution to balance resolution for identifying relevant E–P interactions with sensitivity in loop calling, which improves at lower resolutions. Per-sample loop calling generated, on average, 112,081 unique interactions per sample, ranging from 580 to 436,780. Filtered read pairs from the HiC-Pro pipeline were converted into .hic format files for visualization and normalization^70^.

### WGS analysis

WGS reads were aligned to the hg38 genome using BWA-MEM, and variants were called using the Genomic Data Commons (GDC)/Sanger WGS Variant Calling pipeline (https://docs.gdc.cancer.gov/Data/Bioinformatics_Pipelines/DNA_Seq_Variant_Calling_Pipeline/#whole-genome-sequencing-variant-calling)^71^. Briefly, SNV calls were generated with CaVEMan^72^, small insertions/deletions were identified using Pindel^73^, structural variants were identified using BRASS (https://github.com/cancerit/BRASS) and somatic CN alterations were identified using AscatNGS^74^. WGS read depth statistics were generated using mosdepth (v.0.3.1)^75^. We performed quality control on CN calls (CNVs) generated using the ASCAT pipeline by comparing them with manually reviewed calls from running the ABSOLUTE pipeline on SNP array data. ASCAT does not explicitly output ploidies, so we calculated its estimated ploidy by averaging the total CN of segments weighted by their lengths. For most samples, we observed concordant estimates from both pipelines, and further normalizing CNs by estimated ploidies resolved the majority of discordances. These ploidy-normalized values are used to compare the contribution of CNs to the gene expression across tumors with different ploidy levels. We also examined calls to detect high levels of noise by counting the number of segments and used 1,000 segments to identify hyper-segmented samples. Four samples with associated HiChIP data surpassed this cutoff and were excluded from further analysis. These four samples were associated with cases for which multiple WGS sequencing libraries were generated, and the other WGS library was used for subsequent analysis. To assess the consistency of WGS CNV calls with prior studies, we determined the proportion of cases within each cancer type with either CNV gain (>1 log_2_(ploidy-corrected CNV)) or CNV loss (<−1 log_2_(ploidy-corrected CNV)) in 1 Mb genomic windows.

### HiChIP interaction annotation

We annotated significant HiChIP interactions identified by FitHiChIP based on overlap with gene promoters and/or enhancers. First, we intersected FitHiChIP loop anchors with gene promoters obtained from TxDb.Hsapiens.UCSC.hg38.knownGene (v.3.10.0) and extended by ±1 kb. Anchors that did not overlap with a gene promoter were then intersected with the union H3K27ac peak set to identify anchors that overlap with putative enhancers. HiChIP interactions were then annotated as either E–P, enhancer–enhancer (E–E), promoter–promoter (P–P), enhancer–neither (E–N) or promoter–neither (P–N). Loop classifications were based on annotation of loop anchors, with loop anchors annotated as promoter if they overlapped the promoter (±1 kb of annotated TSS) of at least one gene, enhancer if they overlapped with an H3K27ac peak and no promoters, and neither if they did not overlap with a promoter or H3K27ac peak.

### Interaction matrix visualization

Two-dimensional interaction matrices were visualized using Juicebox (v.1.11.08) or with the plotgardener package in R (v.1.2.10)^76^.

### Eigenvector calculation and A/B compartment annotation

The eigenvector (first principal component of Pearson’s matrix) for H3K27ac HiChiP observed/expected interaction matrices was obtained from .hic files using juicer_tools eigenvector function (v.1.9.9) at 500-kb resolution with Knight–Ruiz (KR) normalization. The sign of the eigenvector and A/B compartment annotation was assigned based on correlation with DNA methylation eigenvector and compartment analysis obtained from additional file 2 of ref. ^26^. A positive eigenvector sign is used to indicate A (open) compartment and a negative sign to indicate B (closed) compartment, the opposite of the eigenvector sign convention used in ref. ^26^, and thus the eigenvector sign is flipped relative to the sign in ref. ^26^.

### H3K27ac 1D signal and virtual 4C visualization

One-dimensional H3K27ac enrichment and ATAC–seq signal were visualized following normalization by reads in TSS regions as described in the ArchR package^77^. ATAC–seq signal tracks were obtained from the GDC publication page^12^. H3K27ac ChIP–seq signal tracks were obtained from ENCODE (accessions ENCFF905FLR and ENCFF873MWG)^28,78^. Virtual 4C plots were generated from dumped matrices generated with Juicer Tools (1.9.9). The Juicer Tools tools dump command was used to extract the chromosome of interest from the .hic file. The interaction profile of a 10-kb bin containing the anchor was then plotted in R (v.4.0.3) after normalization by the total number of valid read pairs and smoothing with the rollmean function from the zoo package (v.1.8-9).

### Generation of union H3K27ac peak and interaction count matrices

One-dimensional H3K27ac peaks called by MACS2 were merged using bedtools merge, and peak signal was calculated using bedtools coverage using 1D H3K27ac signal bed files (v2.28.0). Significant HiChIP interactions identified by FitHiChIP were merged using FitHiChIP’s CombineNearbyInteraction.py, and the loop signal was calculated using pgltools coverage (v.2.2.0)^79^. Raw peak and loop signal were normalized using DESeq2’s size factors normalization obtained using counts(dds,normalized = TRUE) (v.1.30.1)^80^. CNV correction was performed for cases with matching WGS data by dividing normalized signal by ploidy-corrected relative CNV values for peaks or loops overlapping with amplified genomic intervals (relative CNV > 1). Peaks or loops that overlapped genomic intervals with CNV equal to zero or no CNV call were converted to NA values for those samples. For CNV correction of 2D loop signal, the relative CNV value of each loop anchor was determined, and the normalized loop signal was divided by the product of the CNV values at the two anchors. Seven samples did not have matched WGS data for CNV correction and were excluded from further analysis.

### Unsupervised hierarchical clustering and cluster purity calculation

For hierarchical clustering in Fig. 1f, we used CALDER^29^ (v.2.0) to obtain subcompartment calls at 10-kb resolution and performed clustering using vectorized subcompartment annotations based on the compartment rank annotation returned by CALDER. Pairwise Pearson correlations were calculated using the cor function in R using ‘pairwise.complete.obs’. Heatmap visualization and hierarchical clustering were performed using the pheatmap function in R (v.1.0.12). Clustering assignments were obtained using the cutree function in R with *k* equal to the number of unique cancer types. Clustering purity and entropy were calculated using the purity and entropy functions from the NMF package in R (v.0.26)^81^.

For 1D H3K27ac and loop signal clustering, pairwise Pearson correlations were calculated using the normalized, CN-corrected count matrices. Peaks and loops on chrX and chrY and those overlapping hg38 blacklist regions^82^ (https://github.com/Boyle-Lab/Blacklist/blob/master/lists/hg38-blacklist.v2.bed.gz) were excluded from analysis. Correlation analysis was performed on reproducible peaks and loops where at least two samples had a normalized count value ≥3. Count matrices were log_2_-transformed using a prior count of 1 to reduce the contribution of variance from elements with low count values and to avoid taking the log of zero. Visualization, clustering and purity calculations were performed as described above.

### Modeling of oncogene expression with CN and enhancer activity

To determine the relative contributions of CN and enhancer activity to variability in oncogene expression, we integrated H3K27ac peaks and interactions, WGS ploidy-corrected CNV calls and HTSeq counts from RNA-seq data for annotated gene loci. Samples missing from any of these datasets were excluded from this analysis. RNA-seq raw counts were normalized using DESeq2’s size factors normalization obtained using counts(dds,normalized = TRUE) (v.1.26.0). Union H3K27ac peaks within 1 Mb away from annotated gene TSSs that were supported by peak–TSS interaction loops in HiChIP were considered. To account for increased HiChIP read counts due to CNV, read counts of these TSS-associated H3K27ac peaks were normalized to ploidy-corrected CNs as follows: CNV-normalized peak count = (DESeq2-normalized peak count)/(ploidy-corrected CN × 2 + 1). To assess the variability in gene expression, we first filtered on expressed genes defined as genes with more than ten transcripts per million in more than three samples in the RNA-seq dataset. We then used multiple linear regression to model the DESeq2-normalized RNA-seq gene expression values using the formula RNA ~ H3K27ac + CN, where RNA is the DESeq2-normalized RNA-seq gene expression value, H3K27ac represents terms of log_2_-transformed, scaled and centered 1D H3K27ac counts of peaks associated with the given gene and CN represents the ploidy-corrected CN of the gene. For genes with which more than five H3K27ac peaks were associated, log_2_-transformed, scaled and centered 1D H3K27ac counts were reduced to five principal components using the pca function in R with ncomp = 5, center = TRUE, scale = TRUE. For genes with five or less linked H3K27ac peaks, individual peak signal was used as input for RNA expression modeling rather than PCs. Relative importance of model predictors for each gene was quantified with the Lindeman, Merenda and Gold (LMG) method using the calc.relimp function in R with type = ‘lmg’, rela = FALSE. To analyze the relative importance of H3K27ac HiChIP signal and CN of oncogenes, we curated a list of oncogenes and possible oncogenes based on previous analysis^35^. log_2_ transformation of count data was performed as log_2_(count + 1) unless specified otherwise.

### Sample-specific scATAC–seq data analysis

The processed scATAC–seq ArchR object (v1.0.1) with cell-type annotation was obtained from the associated publication^37^. For each sample with matched H3K27ac HiChIP data, we regenerated ArchR object and recalculated chromatin accessibility peaks for each cell population through MACS2 (v2.1.1) under default setting.

### HiChIP integration with scATAC–seq

In total, 29 samples with matched H3K27ac HiChIP and scATAC–seq data were used. A minimum number of 110 noncancer cells was required in each sample to ensure the power of scATAC–seq peak signal detection in the TME, which ends up with 16 samples for integration. For each matched sample, we examined the co-occurrence of H3K27ac peaks and scATAC–seq peaks in the anchor regions of enhancer–promoter interactions. The cell-type-specific enhancer–promoter interaction was identified when (1) the promoter region of the regulated gene had both H3K27ac and scATAC–seq peaks and (2) the enhancer region defined by the HiChIP interactions had H3K27ac peaks but was uniquely accessible in a specific cell type. The cell type shared enhancer–promoter interaction was defined when the promoter or enhancer regions had both H3K27ac and scATAC–seq peaks but were not limited to a specific cell type. The ambiguous enhancer–promoter interaction was defined when both promoter and enhancer region could not map to any scATAC peaks. To generalize our sample-specific analysis to the broader population, we performed a correlation analysis between the enhancer–promoter interaction signal and the corresponding cell fraction in the TME. We obtained these cell fractions from scATAC–seq and estimated leukocyte fractions from RNA-seq data. The Spearman correlation coefficient (Rho) was calculated for each correlation, and we applied cutoff values of Rho ≥0.30 and Rho ≥0.25 to filter the results. For validation of H3K27ac HiChIP deconvolution in TME, the RNA-seq-derived leukocyte fraction estimation, ImmuneScore and tumor purity estimation were downloaded, respectively, from the original publication for correlation analysis^39,40,83^.

### Identification of noncoding mutation involved H3K27ac modification

In total, 62 samples with matched H3K27ac HiChIP and WGS data were used. We used the somatic mutation calling from WGS data as the ground truth. The mutation allele frequency of H3K27ac HiChIP data was generated using bcfools. First, the globally aligned H3K27ac BAM files from the FitHiChIP pipeline were piled up through the mpileup function from bctools (v1.17). Then, the derived BCF files were converted into VCF files through the call function from bcftools. The allele frequency of each somatic mutation was quantified from the VCF files accordingly. The read coverage of H3K27ac HiChIP at the somatic mutation site was calculated through multiBamSummary from deeptools. To ensure accurate allele frequency estimations, we filtered somatic mutations with read counts >30 in both WGS and H3K27ac HiChIP. The significance of the mutant allele was estimated using Fisher’s exact test, followed by the Benjamini–Hochberg (BH) method for multiple comparison correction.

The H3K27ac signal change involved in the mutation site was quantified using the 2-kb window that centered at the mutation position. The 2-kb window was split into 20 bins, with each bin equal to 100 bp. The H3K27ac HiChIP signal was calculated through multiBamSummary from deeptools (v2.0) and normalized by the library size and CN. For each mutation, we performed *t* test between mutant samples and wild-type samples to quantify the difference in CNV-corrected H3K27ac signals. To perform multiple comparison correction, we used the BH method.

### Quantification of noncoding mutation involved motif enrichment changes

chromVARmotifs R package (v0.2) was used for the collection of human TF binding motifs. motifmatchr R package was used for performing motif enrichment analysis. First, a 21-bp sequence centered at mutation position was derived. Then, the matchMotifs function was applied to the 21 bp sequences from mutant and wild type for motif enrichment calculation under the parameter out = ‘positions’ with a *P* value cutoff of 0.01.

### AmpliconArchitect reconstruction of complex structural rearrangements

We collected 120 tumor WGS samples from 15 distinct cancer types and 123 matched normal WGS samples from TCGA, all aligned to GRCh38. We ran AmpliconSuite v.0.931.4 (https://github.com/AmpliconSuite/AmpliconSuite-pipeline), which invoked CNVkit^84^ to call genome-wide CN profiles and identify seed amplicon intervals with CN values larger than 4.5 from these aligned WGS samples. We then ran AmpliconArchitect^60^ v,1.3_r1 to infer the structure of focal amplifications from each sample, with the aligned WGS reads and seed amplicon intervals as input. AmpliconArchitect was run with parameters -insert_sdevs 9 to filter artifactual discordant reads and improve runtime performance and default parameters otherwise. Focal amplifications were classified as cyclic, BFB, complex, linear or invalid using AmpliconClassifier v.0.4.10 (https://github.com/AmpliconSuite/AmpliconClassifier).

### HiChIP visualization at structural rearrangements with NeoLoopFinder

We ran NeoLoopFinder^54^ v.0.2.5 to search for chromatin loops on rearranged genomes (corresponding to local assemblies of linked breakpoints) and CN-corrected H3K27ac HiChIP matrices. Input cool files were generated at 10-kb resolution from .hic files using HiCExplorer’s hicConvertFormat (v.2.2) and balanced using cooler balance (v.0.9.1). ASCAT CNV calls were used for CNV correction using NeoLoopFinder’s correct-cnv, and BRASS SVs were used for complex SV assembly with NeoLoopFinder’s assemble-complexSVs and supplemented with local assemblies from AmpliconArchitect cycle decomposition. Neoloops were detected using neoloop-caller -O neo-loops.txt allValidPairs.cool --assembly assemblies.txt --balance-type CNV --protocol insitu --prob 0.95 --nproc 20.

### Reporting summary

Further information on research design is available in the Nature Portfolio Reporting Summary linked to this article.

## Online content

Any methods, additional references, Nature Portfolio reporting summaries, source data, extended data, supplementary information, acknowledgements, peer review information; details of author contributions and competing interests; and statements of data and code availability are available at 10.1038/s41588-025-02188-0.

## Supplementary information

Supplementary Information Supplementary Methods.

Reporting Summary

Supplementary Tables Supplementary Tables 1–9.

## Data Availability

Processed data not provided in the supplementary data files are available through the TCGA Publication Page (https://gdc.cancer.gov/about-data/publications/TCGA-HiChIP-2024). Raw HiChIP data as fastq files are available through the NIH Genomic Data Commons portal (https://portal.gdc.cancer.gov/), and accession information is available on the TCGA Publication Page.

## Code availability

Custom code used in this study is available at https://github.com/NCICCGPO/HiChIP-Manuscript and via Zenodo at 10.5281/zenodo.15103075 (ref. ^85^).
