# Supplementary material for: Three-dimensional genome landscape of primary human cancers
Source: Nat Genet. 2025 May 12;57(5):1189–200. doi: 10.1038/s41588-025-02188-0 (PMC12081301; doi:10.1038/s41588-025-02188-0)
Supplement: Supplementary file 1 — Supplementary Methods. [file 41588_2025_2188_MOESM1_ESM.pdf]

nature genetics

Supplementary information

https://doi.org/10.1038/s41588-025-02188-0

Three-dimensional genome landscape of primary human cancers

In the format provided by the authors and unedited

Supplementary Methods

Nuclei isolation detailed protocol

Nuclei were isolated from 20 mg frozen tumor tissue by douncing with 1 mL cold 1X Homogenization Buffer (HB; 250 mM sucrose, 25 mM KCl, 5 mM $MgCl_2$, 20 mM Tricine-KOH pH 7.8, 1 mM DTT, 500 uM Spermidine, 150 uM Spermine, 0.3% NP40, 1X cOmplete Protease Inhibitor). Homogenate was filtered using a 70 um Flowmi strainer and nuclei pelleted by spinning 5 min at 4°C at 350 rcf. Nuclei were resuspended in 400 uL of 1X HB before adding 400 uL of 50% Iodixanol Solution (50% Iodixanol, 25 mM KCl 5 mM $MgCl_2$, 20 mM Tricine-KOH pH 7.8) and mixed well by pipetting. 600 uL of 30% Iodixanol Solution (30% Iodixanol, 25 mM KCl 5 mM $MgCl_2$, 20 mM Tricine-KOH pH 7.8) was layered under the resulting 25% mixture, and 600 uL of 40% Iodixanol Solution (40% Iodixanol, 25 mM KCl 5 mM $MgCl_2$, 20 mM Tricine-KOH pH 7.8) was layered under the 30% mixture. Nuclei were spun for 20 min at 4°C at 3,000 rcf in a swinging bucket centrifuge with the brake off. The nuclei band at the 30-40% interface was transferred to a fresh tube and diluted with ATAC-RSB-Tween Buffer (10 mM Tris-HCl pH 7.5, 10 mM NaCl, 3 mM $MgCl_2$, 0.1% Tween-20). Nuclei were spun down by centrifugation for 10 min at 500 rcf at 4°C and resuspended in cold BAM Banker (Wako Chemicals) for cryopreservation. An up-to-date version of this protocol is maintained on protocols.io (https://www.protocols.io/view/isolation-of-nuclei-from-frozen-tissue-for-snmulti-kxygxmr34l8j/).

HiChIP library generation detailed protocol

<u>Crosslinking</u>

One million cryopreserved nuclei were used for HiChIP library generation. Nuclei were fixed with 1 mL of 1% formaldehyde in PBS for 10 minutes at room temperature with rotation. Formaldehyde was quenched with 110 uL 1.25 M glycine for a final concentration of 125 mM glycine and incubated for 5 minutes at room temperature with rotation. After quenching, 1 volume (1.11 mL) cold PBS + 0.2% Tween-20 was added and fixed nuclei pelleted by centrifugation at 2000 rcf at 4°C for 5 minutes.

<u>Lysis and Restriction Digest</u>

Crosslinked nuclei were resuspended in 500 uL cold Hi-C Lysis Buffer (10 mM Tris-HCl pH 8.0, 10 mM NaCl, 0.2% NP-40, 1X protease inhibitor (Roche)) and incubated at 4°C for 30 minutes with rotation. Lysates were centrifuged at 2500 rcf at 4°C for 5 minutes, pelleted nuclei resuspended in 500 uL cold Hi-C Lysis Buffer, and centrifuged again at 2500 rcf at 4°C for 5 minutes. Nuclei pellet was resuspended in 95 uL water before adding 5 uL 10% SDS, mixed

gently by pipetting, and incubated at 62°C without shaking for 10 minutes. SDS was quenched by adding 285 uL water and 50 uL 10% Triton X-100, mixed gently by inverting and incubated at 37°C for 15 minutes without shaking. Restriction digest was performed by adding 50 uL of 10X CutSmart Buffer (NEB), 4 uL of MboI enzyme (100 U total, 25 U/μL, NEB), 11 uL of water, and digested for 15 minutes at 37°C with rotation in a hybridization oven. Nuclei were pelleted by adding 5 uL 10% Tween-20, mixing well, and centrifuging at 2500 rcf for 5 minutes at 4°C. Nuclei were washed with 1000 uL of cold 1X CutSmart buffer (NEB) containing 0.1% Tween-20 and centrifuged at 2500 rcf for 5 minutes at 4°C. Nuclei were resuspended in 500 uL of cold 1.1X CutSmart buffer (NEB).

Incorporation and Proximity Ligation

To fill in restriction fragment overhangs and mark the DNA ends with biotin, 50 uL of fill-in master mix was added (15 uL 1 mM biotin-dATP (Thermo), 4.5 uL 10 mM dCTP/dGTP/dTTP mix (3.33 mM each), 10 uL 5 U/uL DNA Polymerase I, Large (Klenow) Fragment (NEB), 20.5 uL water). Reactions were mixed and incubated at 37°C for 15 minutes with rotation in a hybridization oven. After ligation, 948 uL of ligation master mix was added (150 uL 10X T4 DNA ligase buffer (NEB), 125 uL 10% Triton X-100, 3 uL 50 mg/mL BSA, 10 uL 400 U/uL T4 DNA Ligase (NEB), 660 uL water) and incubated at room temperature for 2 hours with rotation. Nuclei were pelleted at 2500 rcf for 5 minutes at room temperature.

Sonication

For sonication, nuclei were resuspended in 880 uL Nuclear Lysis Buffer (50 mM Tris-HCl pH 7.5, 10 mM EDTA, 1% SDS, 1X protease inhibitor (Roche)) and transferred to a Covaris millitube and sheared in a Covaris E220 with the following parameters: fill level = 10, duty cycle = 5, PIP = 140, cycles/burst = 200, time = 4 min and then clarified by centrifugation for 15 min at 21,000 rcf at 4°C.

Immunoprecipitation

For immunoprecipitation, clarified supernatant was transferred to a pre-chilled 15 mL conical tube and 3X volume (2640 uL) cold ChIP Dilution Buffer added (0.01% SDS, 1.1% Triton X-100, 1.2 mM EDTA, 16.7 mM Tris pH 7.5, 167 mM NaCl). 2 ug of H3K27ac antibody (Abcam ab4729) was added and incubated at 4°C overnight with rotation. 30 uL Protein A beads per sample were washed twice with 1000 uL ChIP Dilution Buffer using a magnetic tube rack. Protein A beads were resuspended in 100 uL ChIP Dilution Buffer per sample, added to sample, and rotated at 4°C for

2 hours. Bead washes were performed using magnetic separation and washed three times each with 500 uL cold Low Salt Buffer (0.1% SDS, 1% Triton X-100, 2 mM EDTA, 20 mM Tris-HCl pH 7.5, 150 mM NaCl), High Salt Wash Buffer (0.1% SDS, 1% Triton X-100, 2 mM EDTA, 20 mM Tris-HCl pH 7.5, 500 mM NaCl), and LiCl Wash Buffer (1 mM EDTA, 10 mM Tris-HCl pH 7.5, 250 mM LiCl, 1% NP-40, 1% sodium deoxycholate). Immunoprecipitated DNA was eluted from beads by adding 100 uL DNA Elution Buffer (50 mM Tris pH 8.0, 1% SDS) and incubating at room temperature for 10 minutes with rotation followed by 3 minutes at 37°C with 1400 rpm shaking. Supernatant was removed using magnetic separation and elution repeated with an additional 100 uL of DNA Elution Buffer. 10 uL of Proteinase K (20 mg/mL) was added to elution and incubated at 55°C for 45 minutes with 1400 rpm shaking, followed by 1.5 hours at 67°C with 1400 rpm shaking. Immunoprecipitated DNA was purified using ChIP Clean and Concentrator column purification (Zymo) and eluted in 21 uL of water. Post ChIP DNA was quantified by Qubit and a maximum of 100 ng used for biotin capture.

Biotin Pull-Down and Preparation for Illumina Sequencing

For each sample, 10 uL of Streptavidin C-1 beads were washed twice with 1 mL of Tween Wash Buffer (5 mM Tris-HCl pH 7.5, 0.5 mM EDTA, 1M NaCl, 0.05% Tween-20) using magnetic separation. Washed beads were resuspended in 20 uL of 2X Biotin Binding Buffer (10 mM Tris-HCl pH 7.5, 1mM EDTA, 2M NaCl), added to 20 uL of post ChIP DNA sample, and incubated at room temperature for 15 minutes with gentle vortex mixing. Beads were separated on a magnet and washed twice with 500 uL Tween Wash Buffer at 55°C for 2 minutes with 1400 rpm shaking. Beads were washed on a magnet with 200 uL of cold 1X TD buffer (10 mM Tris-HCl pH 7.5, 5 mM $MgCl_2$, 10% dimethylformamide) and mixed by rotation before discarding the supernatant. Beads were resuspended in 50 uL cold 1X TD Buffer with Tn5 enzyme (Illumina), with 0.5 uL of 1:20 diluted Tn5 (in 1X TD Buffer) used per ng of post-ChIP DNA. DNA was transposed at 55°C with interval shaking at 1400 rpm for 10 minutes. After transposition, beads were separated on a magnet to remove the supernatant and 200 uL of 55°C 50 mM EDTA added followed by incubation at 55°C for 30 minutes with 1400 rpm shaking. Beads were washed twice with 500 uL pre-heated 55°C Tween Wash Buffer with incubation at 55°C for 2 minutes with 1400 rpm shaking. After washes, beads were resuspended in 200 uL room temperature 10 mM Tris pH 8.0.

PCR and Post-PCR Size Selection

Beads were resuspended in 50 uL PCR Master Mix (1X Phusion HF, 250 nM Nextera Ad1.1 universal primer, and 250 nM Nextera Ad2.X barcoded primer) and amplified using the following

cycling conditions: 72°C for 5 min, 98°C for 1 min, then 6-9 cycles [98°C for 15 s, 63°C for 30 s, and 72°C for 1 min], followed by 72°C for 3 min. Primer sequences are listed in **Supplementary Table 9**. Cycle number was determined based on post-ChIP DNA yield (6.25-12.49 ng DNA = 9 cycles, 12.5-24.9 ng DNA = 8 cycles, 25-49.9 ng DNA = 7 cycles, 50-100 ng DNA = 6 cycles). Amplified libraries were size-selected using either PAGE gel size selection after cleanup with DNA Clean and Concentrator column (Zymo) or two-sided size selection with Ampure XP beads (0.5X followed by 0.8X). Libraries were quantified using qPCR, diluted to 4 nM and sequenced on an Illumina HiSeq 4000 with paired-end 75 bp reads.

WGS cluster amplification and sequencing

Following sample preparation, libraries are quantified using quantitative PCR (kit purchased from KAPA Biosystems), with probes specific to the ends of the adapters. This assay is automated using Agilent's Bravo liquid handling platform. Based on qPCR quantification, libraries are normalized to 2.2nM and pooled into 24-plexes. Sample pools are combined with NovaSeq Cluster Amp Reagents DPX1, DPX2 and DPX3 and loaded into single lanes of a NovaSeq 6000 S4 flowcell cell using the Hamilton Starlet Liquid Handling system. Cluster amplification and sequencing occur on NovaSeq 6000 Instruments utilizing sequencing-by-synthesis kits to produce 151bp paired-end reads. Fastqs were processed by the Picard data-processing pipeline to yield CRAM or BAM files containing demultiplexed, aggregated aligned reads. All sample information tracking is performed by automated LIMS messaging.

HiChIP data QC - Transcription start site enrichment

Enrichment of H3K27ac HiChIP signal at transcription start sites (TSSs) was used to quantify H3K27ac ChIP enrichment quality, similar to ATAC-seq quality control[1]. First, allValidPairs generated by HiC-Pro were read into a GenomicRanges object in R. Pairs separated by more than 10 kb were excluded. TSSs were obtained from TxDb.Hsapiens.UCSC.hg38.knownGene (version 3.10.0) and extended 2000 bp in each direction and overlapped with fragments (both ends of a valid pair) using GenomicRange's findOverlaps. Next, the distance between the fragments and the strand-corrected TSS was calculated and the number of fragments occurring in each single-base bin was summed. To normalize this value to the local background, the enrichment at each position +/- 2000 bp from the TSS was normalized to the mean of the enrichment at positions +/-1900-2000 bp from the TSS. The final TSS enrichment reported was the maximum enrichment value within +/- 50 bp of the TSS after smoothing with a rolling mean every 51 bp.

HiChIP data QC - genotype correlation with TCGA SNP array data

In order to validate the authenticity of HiChIP data attributed to specific TCGA donors and their corresponding tissues, we conducted genotyping analyses. Our approach involved comparing our HiChIP data (N=69 individual sequencing experiments) with SNP calls extracted from TCGA SNP array data utilizing the Affymetrix SNP 6.0 array (N=11,127 TCGA donors). This SNP array data, having been previously generated by TCGA, serves as our benchmark for validation. To achieve this, we overlapped genomic locations probed by the Affymetrix SNP 6.0 array (932,148 hg38-mappable probes) with peak regions identified in all HiChIP samples. The genotypic information for each HiChIP BAM file was then collected at 124,773 SNP locations and converted into a birdseed-style format. Notably, a minimum read depth of 6 was set as a prerequisite for SNP calls. In the HiChIP data, positions were labeled as homozygous if reads mapped exclusively to either the A or B allele, resulting in a birdseed call of 0 or 2. Conversely, positions were categorized as heterozygous if the absolute difference between allele A and allele B counts was less than 50% of the total depth, leading to a birdseed value of 1. Positions exhibiting substantial allelic imbalance were classified as homozygous due to excessive disparity, with a birdseed value of 0 or 2. Each birdseed-style HiChIP genotyping list was correlated with TCGA Affymetrix SNP 6.0 array data (11,127 individual donors). Pearson correlations were computed solely for HiChIP BAM files at genomic locations with a viable SNP call in the HiChIP data (locations with read depth exceeding 6). Samples were considered successful if their correlation with the expected biological donor surpassed the correlation with all other 11,126 TCGA donors, affirming concordance between HiChIP data and Affymetrix SNP 6.0 array data, and thereby validating their shared origin.

H3K27ac peak annotation

Merged H3K27ac peaks were annotated using HOMER's annotatePeaks.pl (version 4.11)[2]. H3K27ac HiChIP 1D peaks were overlapped with ENCODE H3K27ac ChIP-seq peaks obtained from MACS narrowPeak files from primary tissue samples with accession numbers listed in Supplementary Table 8. Number of interacting gene promoters with H3K27ac peaks and number of genes skipped by loops were determined using GenomicRanges' findOverlaps function (version 1.42.0) with gene promoters obtained from TxDb.Hsapiens.UCSC.hg38.knownGene (version 3.10.0).

Comparison to HiChIPdb loops

10kb resolution FitHiChIP loops from H3K27ac HiChIP experiments were downloaded from HiChIPdb[3]. hg19 coordinates were converted to hg38 using the easyLiftOver function from the R package easyLift (https://github.com/caleblareau/easyLift, version 0.2.1). Loop sets were converted to GenomicInteractions format in R (version 1.24.0) and the intersection between HiChIPdb loops and our loop set was determined using GenomicRanges' findOverlaps function (version 1.42.0).

Cluster purity and entropy calculations

Clustering purity and entropy were calculated using the purity and entropy functions from the NMF package in R (version 0.26)[4]. Cluster purity is calculated as

$$Purity = \frac{1}{n}\sum_{q=1}^{k} \quad max_{1\leq j\leq l} n_q^j ,$$

where n is the total number of samples, n_q^j is the number of samples in cluster q that belongs to original class j $(1 \leq j \leq l)$. Cluster purity quantifies the degree that cells of the same cancer type cluster together, calculated by counting the number of samples belonging to the most common cancer type assigned to each cluster, summing across clusters, and dividing by the total number of samples. Cluster entropy is calculated as

$$Entropy = -\frac{1}{n\, log_2 l}\sum_{q=1}^{k} \sum_{j=1}^{l} n_q^j log_2 \frac{n_q^j}{n_q}$$

where n is the total number of samples, n_q is the total number of samples in cluster q $(1 \leq q \leq k)$, and n_q^j is the the number of samples in cluster q that belongs to original class j $(1 \leq j \leq l)$. The smaller the entropy, the better the clustering performance.

Dimensionality reduction with t-SNE

Count matrices used for unsupervised hierarchical clustering were used for dimensionality reduction and visualization using t-Distributed Stochastic Neighbor Embedding (t-SNE). Log-transformed counts were scaled using Seurat's ScaleData and element counts were ranked by variance using matrixStats rowVars function. The top 10,000 variable elements were used for principal component analysis (PCA) using Seurat's RunPCA function. The top 15 PCs were used for t-SNE dimensionality reduction using Seurat's RunTSNE with perplexity = 5. Samples were colored by cancer type, bulk ATAC-seq cluster annotation[1], BRCA subtype[5], and ESCA subtype[6].

Identification of differential H3K27ac peaks and HiChIP loops by feature binarization

We executed the identification of 'unique' peaks within the HiChIP data, adhering to a predefined methodology. In essence, we $\log_2$-transformed the copy number corrected H3K27ac peak count matrix, categorizing individual cancer types as distinct 'groups'. For each peak within the HiChIP peak set, we computed both intragroup mean and standard deviation values. Subsequently, these groups were ranked based on their respective intragroup mean scores. Through an iterative process, we initiated from the second-lowest-ranked group and gauged whether its mean value surpassed the sum of the maximum intragroup mean and the intragroup standard deviation of the subsequent-lower group. This iterative sequence persisted until a group meeting this particular criterion was identified. This point defined the 'breakpoint'. Groups boasting intragroup means exceeding the breakpoint were labeled '1' for that specific peak, while groups situated below the breakpoint received a '0' designation. Peaks lacking a breakpoint were excluded. This 'binarization' process established all '1s' as being greater than any individual '0', thus capturing peaks unique to multiple groups. Combinations present in three or fewer groups were retained. To address multiple hypothesis testing, we devised a contrast matrix for observed combinations and subjected the log-normalized counts matrix to limma's (v.3.38.3) eBayes test. Subsequently, we extracted false discovery rate (FDR)-adjusted P values from differential testing, preserving peaks with FDR values below 0.01. Employing the same aforementioned methodology, we also determined the 'unique' interactions within the HiChIP data by using the $\log_2$-transformed copy number corrected H3K27ac interaction count matrix. For motif enrichment analysis, we transformed the 'unique' peaks of each cancer type into the bed format. However, due to the vast genomic span covered by 'unique' interactions, conducting direct motif enrichment analysis proved challenging. As a solution, we intersected the 'unique' interactions per cancer type with the corresponding H3K27ac peaks. Peaks overlapping both anchors were consolidated into the bedgraph format to facilitate motif enrichment analysis. The findMotifsGenome function from HOMER software (v4.11.1) was employed for this purpose, using the parameter '-size given'.

Enhancer rewiring analysis

Using the normalized and copy number corrected consensus FitHiChIP loops from H3K27ac HiChIP 3D data, we intersected the loop anchor with consensus peaks from H3K27ac 1D data as well as promoters of gene transcripts. Promoters are defined as -2500/+250 bp of each TSS using GENCODE v36. In situations where the peak is involved in a given peak-promoter interaction in one sample but not called as a peak by H3K27ac 1D in that sample, "0" will be assigned to the peak-promoter interaction for that given sample. We also focused on enhancer-promoter

interactions by excluding H3K27ac 1D peaks overlapping any promoters when interacting with another promoter. Overall, from consensus loops with 10kb anchors, we identified 894,776 enhancer-promoter interactions.

Oncogene enhancer usage classification

To classify oncogene enhancer usage as dynamic, selective, or static, we compared the average loop variance per oncogene between cancer types versus maximum $\log_2$ fold change across all loops linked to a given gene. Oncogenes with average variance > 10 were classified as dynamic, genes with maximum $\log_2$ fold change >= 1.5 and average variance < 10 classified as selective, and genes with maximum $\log_2$ fold change < 1.5 and average variance < 10 classified as static. Given the continuum between static and selective enhancer usage based on maximum $\log_2$ fold change, a cutoff of 1.5 was chosen to reflect the average maximum $\log_2$ fold change across all oncogenes.

HiChIP integration with cancer associated SNP sites

Cancer-associated SNP data were retrieved from the database available at https://www.ebi.ac.uk/gwas/. We augmented the SNP list by incorporating SNPs in high Linkage Disequilibrium (LD) with GWAS lead SNPs (LD r2 > 0.8). This LD data was sourced from the haploreg website (http://archive.broadinstitute.org/mammals/haploreg/data/). To identify potential regulatory elements associated with these SNPs, we performed an intersection analysis with enhancer peaks. The enhancer peaks were obtained from malignant cell-specific promoter-enhancer interactions, as determined through our prior HiChIP decomposition analysis. This approach allowed us to pinpoint genomic positions where cancer-associated SNPs coincided with enhancer elements.

AmpliconArchitect reconstruction of complex structural rearrangements

AmpliconArchitect (AA)[7] version 1.3_r1 was used to infer the structure of focal amplifications from each sample, with the aligned WGS reads and seed amplicon intervals as input. A focal amplification is composed of a collection of genomic segments connected by breakpoints indicating either a CN change between two consecutive segments, or a rearrangement connecting two nonadjacent segments. A single sample can contain multiple non-overlapping focal amplifications. AA represents focal amplifications in the form of a copy-number aware breakpoint graph, where nodes represent genome segments and edges represent junctions between segments, including breakpoint connections. AA further decomposes the breakpoint

graph into a collection of cyclic and non-cyclic paths, each representing a potential structure or substructure (i..e, local assembly) comprised of genome segments connected by a chain of breakpoints. The structural signatures in these paths are subsequently used to classify the type of focal amplification.

Amplicon classification

We ran AmpliconClassifier version 0.4.10 (https://github.com/AmpliconSuite/AmpliconClassifier) using the AA-derived breakpoint graph and cycles files to classify each focal amplification into five categories: (1) cyclic amplification (potential ecDNAs); (2) BFB amplification; (3) Complex non cyclic amplification; (4) Linear amplification; and (5) Invalid focal amplification. We summarize the AmpliconClassifer rules (originally described in Kim et al. and Luebeck et al.[8,9]) as follows. As a prerequisite, focal amplifications must contain ≥10 kb of total genomic segments amplified to at least 5 copies above median ploidy to be considered valid. Focal amplifications were classified as BFB if they met the criteria for a BFB amplification (i.e., if breakpoints representing foldback events account for at least 25% of all SVs in the amplicon, and the cycles containing a foldback account for at least 60% of the length-weighted total CN of valid amplicon paths decomposed by AA). Focal amplifications not classified as BFB were classified as cyclic if there exists a cycle in the breakpoint graph (representing a potential ecDNA structure), and the total copy counts from cycles account for at least 12% of the total length-weighted CN. Acyclic focal amplifications were classified as complex non cyclic if they contained at least 5 breakpoint edges representing rearrangements, suggesting higher-order rearrangements beyond simple indel SV events. All other valid acyclic focal amplifications were classified as linear. We then hierarchically classified samples based on which type of focal amplifications were present in the sample, giving precedence to cyclic, followed by BFB, complex and linear. For example, a sample with both cyclic and complex focal amplifications would be classified as cyclic. Samples without any valid focal amplifications were similarly classified as 'no focal somatic CN amplification detected'.

HiChIP visualization at structural rearrangements with NeoLoopFinder

NeoLoopFinder[10], by default, computes a genome-wide CN profile and a collection of CN segments from an input HiChIP matrix, and then balances the matrix with a modified ICE procedure by taking the CN segments as input. We provided the NeoLoopFinder pipeline with CN segments estimated from the corresponding WGS samples (based on ASCAT CNV calls) as its input of the CN-aware matrix balancing procedure with NeoLoopFinder's correct-cnv. Given a list of candidate SVs (potentially from other sources, e.g., WGS or OM), NeoLoopFinder then

reconstructs local assemblies representing a chain of one or more SVs from the input list, by shifting or flipping the submatrices according to the coordinates and orientations of the SVs. Therefore, we supplied NeoLoopFinder with a collection of SV breakpoints identified by BRASS from WGS data, which were filtered and used for complex SV assembly with NeoLoopFinder's assemble-complexSVs. In case NeoLoopFinder missed true assemblies, we additionally augmented the assemblies constructed by NeoLooFinder with the collection of local assemblies from AA cycle decomposition as follows. Because NeoLoopFinder does not accept assemblies with duplicated segments, we broke each cycle returned by AA into all possible longest paths of at least 2 non-overlapping segments. We provided these paths as input to NeoLoopFinder to search for chromatin loops in addition to the local assemblies constructed above using neoloop-caller -O neo-loops.txt allValidPairs.cool --assembly assemblies.txt --balance-type CNV --protocol insitu --prob 0.95 --nproc 20. The output of NeoLoopFinder consists of two types of interactions: 'loops,' which represent interactions on a single genomic segment, and 'neo-loops,' representing interactions on two different genomic segments, brought together by an SV. We postprocessed the loops and neoloops identified by NeoLoopFinder in each HiChIP sample (as case sample) by filtering out those that also occur in any other samples without focal amplifications on the same genomic segments (as control samples). In control samples, loops were searched on the same collection of local assemblies as used in the case sample. For comparing the number of loops per classification type, we dropped focal amplifications with total size less than 500kb, which often lead to unreliable classifications, as well as insufficient number of neighboring bins for loop finding.

Co-amplification frequency analysis across TCGA WGS

To identify potential enhancer regions co-focally-amplified with an oncogene of interest (for example, amplified on ecDNA), we binned each focally amplified genome with 10 kb resolution in accordance with HiChIP, and counted the number of samples co-amplified with the given oncogene per bin. Due to small cohort size (243 samples in total), the oncogene of interest is often amplified in very few samples. We overcome this limitation by counting, in each 10kb bin, the number of samples co-amplified with the given oncogene within a larger cohort of 1538 WGS samples from Kim et al[8]. We computed an empirical P-value of co-amplification for each 10kb bin connected with the given oncogene by a loop or neoloop as follows: Let n_0 be the number of samples where bin b_i is co-amplified with gene g. To compute an empirical permutation based p-value, we generated 10,000 datasets randomly shuffling the focally amplified bins in each sample, such that (i) the distance between the first and last amplified bins after shuffling is *at most* that

distance in the original amplification; (ii) the number of contiguously amplified intervals after shuffling *remains the same as* the number in the original amplification; and (iii) bins involving *g* are always amplified. The empirical p-value was given by the fraction of times bin b_i was co-amplified with *g* in at least n_0 samples. Finally, empirical P-values were adjusted for multiple comparisons using the Benjamini-Hochberg procedure.

References

1. Corces, M. R. *et al.* The chromatin accessibility landscape of primary human cancers. *Science* **362**, eaav1898 (2018).
2. Heinz, S. *et al.* Simple combinations of lineage-determining transcription factors prime cis-regulatory elements required for macrophage and B cell identities. *Mol Cell* **38**, 576–589 (2010).
3. Zeng, W., Liu, Q., Yin, Q., Jiang, R. & Wong, W. H. HiChIPdb: a comprehensive database of HiChIP regulatory interactions. *Nucleic Acids Research* **51**, D159–D166 (2023).
4. Gaujoux, R. & Seoighe, C. A flexible R package for nonnegative matrix factorization. *BMC Bioinformatics* **11**, 367 (2010).
5. Sanchez-Vega, F. *et al.* Oncogenic Signaling Pathways in The Cancer Genome Atlas. *Cell* **173**, 321-337.e10 (2018).
6. Cancer Genome Atlas Research Network *et al.* Integrated genomic characterization of oesophageal carcinoma. *Nature* **541**, 169–175 (2017).
7. Deshpande, V. *et al.* Exploring the landscape of focal amplifications in cancer using AmpliconArchitect. *Nat Commun* **10**, 1–14 (2019).
8. Kim, H. *et al.* Extrachromosomal DNA is associated with oncogene amplification and poor outcome across multiple cancers. *Nat Genet* **52**, 891–897 (2020).
9. Luebeck, J. *et al.* Extrachromosomal DNA in the cancerous transformation of Barrett's oesophagus. *Nature* **616**, 798–805 (2023).

10. Wang, X. *et al.* Genome-wide detection of enhancer-hijacking events from chromatin interaction data in rearranged genomes. *Nat Methods* **18**, 661–668 (2021).
