# Supplementary material for: Three-dimensional genome landscape of primary human cancers
Source: Nat Genet. 2025 May 12;57(5):1189–200. doi: 10.1038/s41588-025-02188-0 (PMC12081301; doi:10.1038/s41588-025-02188-0)
Supplement: Supplementary file 2 — Reporting Summary [file 41588_2025_2188_MOESM2_ESM.pdf]

nature portfolio

Corresponding author(s): Howard Y. Chang, William J. Greenleaf

Last updated by author(s): Feb 13, 2025

Reporting Summary

Nature Portfolio wishes to improve the reproducibility of the work that we publish. This form provides structure for consistency and transparency in reporting. For further information on Nature Portfolio policies, see our Editorial Policies and the Editorial Policy Checklist.

Statistics

For all statistical analyses, confirm that the following items are present in the figure legend, table legend, main text, or Methods section.

n/a	Confirmed	
☐	☒	The exact sample size (*n*) for each experimental group/condition, given as a discrete number and unit of measurement
☐	☒	A statement on whether measurements were taken from distinct samples or whether the same sample was measured repeatedly
☐	☒	The statistical test(s) used AND whether they are one- or two-sided<br>*Only common tests should be described solely by name; describe more complex techniques in the Methods section.*
☒	☐	A description of all covariates tested
☐	☒	A description of any assumptions or corrections, such as tests of normality and adjustment for multiple comparisons
☐	☒	A full description of the statistical parameters including central tendency (e.g. means) or other basic estimates (e.g. regression coefficient) AND variation (e.g. standard deviation) or associated estimates of uncertainty (e.g. confidence intervals)
☐	☒	For null hypothesis testing, the test statistic (e.g. *F*, *t*, *r*) with confidence intervals, effect sizes, degrees of freedom and *P* value noted<br>*Give P values as exact values whenever suitable.*
☒	☐	For Bayesian analysis, information on the choice of priors and Markov chain Monte Carlo settings
☒	☐	For hierarchical and complex designs, identification of the appropriate level for tests and full reporting of outcomes
☐	☒	Estimates of effect sizes (e.g. Cohen's *d*, Pearson's *r*), indicating how they were calculated

Our web collection on statistics for biologists contains articles on many of the points above.

Software and code

Policy information about availability of computer code

Data collection

HiChIP data were processed as described previously. In brief, paired-end reads were aligned to the hg38 genome using the HiC-Pro pipeline (v.2.11.0). Default settings were used to remove duplicate reads, assign reads to MboI restriction fragments, filter for valid interactions and generate binned interaction matrices.

WGS sequencing reads were aligned to the hg38 genome using bwa-mem (version 0.7.15).

Data analysis

HiChIP data analysis
FitHiChIP (v.8.0) were used to identify loops. Dangling end, self-circularized, and re-ligation read pairs were merged with valid read pairs to create a one-dimensional H3K37ac signal bed file, corresponding to H3K27ac ChIP-seq-like signal which was used for peak calling and 1D signal quantification using standard ChIP-seq analysis tools including MACS2 (v2.2.7.1). FitHiChIP was used to identify 'peak-to-all' interactions at 10-kb resolution using peaks called from the one-dimensional HiChIP data using MACS2. A lower distance threshold of 20kb was used. Bias correction was performed using coverage specific bias. HiChIP loop calling was performed at 10kb resolution to balance resolution for identifying relevant enhancer-promoter interactions with sensitivity in loop calling which improves at lower resolutions. Per-sample loop calling generated on average 112,081 unique significant interactions per sample, ranging from 580 to 436,780. Filtered read pairs from the HiC-Pro pipeline were converted into .hic format files for visualization and normalization.

WGS analysis
WGS variants were called using the GDC/Sanger Whole Genome Sequencing Variant Calling pipeline (https://docs.gdc.cancer.gov/Data/Bioinformatics_Pipelines/DNA_Seq_Variant_Calling_Pipeline/#whole-genome-sequencing-variant-calling). Briefly, SNV calls were generated with CaVEMan (version 1.15.5), small insertions/deletions identified using Pindel (version 2.0), structural variants identified using BRASS (https://github.com/cancerit/BRASS, version 6.2.1), and somatic copy number alterations identified using AscatNGS (version 4.2.1). WGS read

depth statistics were generated using mosdepth (version 0.3.1). We performed quality control on copy number calls (CNVs) generated using ASCAT pipeline by comparing with manually-reviewed calls from running ABSOLUTE (version 1.0.6) pipeline on SNP array data.

HiChIP data QC - Transcription start site enrichment

Enrichment of H3K27ac HiChIP signal at transcription start sites (TSSs) was used to quantify H3K27ac ChIP enrichment quality, similar to ATAC-seq quality control (Corces et al., 2018). First, allValidPairs generated by HiC-Pro were read into a GenomicRanges object in R. Pairs separated by more than 10 kb were excluded. TSSs were obtained from TxDb.Hsapiens.UCSC.hg38.knownGene (version 3.10.0) and extended 2000 bp in each direction and overlapped with fragments (both ends of a valid pair) using GenomicRange's findOverlaps. Next, the distance between the fragments and the strand-corrected TSS was calculated and the number of fragments occurring in each single-base bin was summed. To normalize this value to the local background, the enrichment at each position +/- 2000 bp from the TSS was normalized to the mean of the enrichment at positions +/-1900-2000 bp from the TSS. The final TSS enrichment reported was the maximum enrichment value within +/- 50 bp of the TSS after smoothing with a rolling mean every 51 bp.

HiChIP data QC - genotype correlation with TCGA SNP array data

In order to validate the authenticity of HiChIP data attributed to specific TCGA donors and their corresponding tissues, we conducted genotyping analyses. Our approach involved comparing our HiChIP data (N=69 individual sequencing experiments) with SNP calls extracted from TCGA SNP array data utilizing the Affymetrix SNP 6.0 array (N=11,127 TCGA donors). This SNP array data, having been previously generated by TCGA, serves as our benchmark for validation. To achieve this, we overlapped genomic locations probed by the Affymetrix SNP 6.0 array (932,148 hg38-mappable probes) with peak regions identified in all HiChIP samples. The genotypic information for each HiChIP BAM file was then collected at 124,773 SNP locations and converted into a birdseed-style format. Notably, a minimum read depth of 6 was set as a prerequisite for SNP calls. In the HiChIP data, positions were labeled as homozygous if reads mapped exclusively to either the A or B allele, resulting in a birdseed call of 0 or 2. Conversely, positions were categorized as heterozygous if the absolute difference between allele A and allele B counts was less than 50% of the total depth, leading to a birdseed value of 1. Positions exhibiting substantial allelic imbalance were classified as homozygous due to excessive disparity, with a birdseed value of 0 or 2. Each birdseed-style HiChIP genotyping list was correlated with TCGA Affymetrix SNP 6.0 array data (11,127 individual donors). Pearson correlations were computed solely for HiChIP BAM files at genomic locations with a viable SNP call in the HiChIP data (locations with read depth exceeding 6). Samples were considered successful if their correlation with the expected biological donor surpassed the correlation with all other 11,126 TCGA donors, affirming concordance between HiChIP data and Affymetrix SNP 6.0 array data, and thereby validating their shared origin.

Interaction and H3K27ac peak annotation

We annotated significant HiChIP interactions identified by FitHiChIP based on overlap with gene promoters and/or enhancers. First we intersected FitHiChIP loop anchors with gene promoters obtained from TxDb.Hsapiens.UCSC.hg38.knownGene (version 3.10.0) and extended by +/- 1kb. Anchors that did not overlap with a gene promoter were then intersected with the union H3K27ac peak set to identify anchors that overlap with putative enhancers. HiChIP interactions were then annotated as either E-P: enhancer-promoter, E-E: enhancer-enhancer, P-P: promoter-promoter, E-N: enhancer-neither, P-N: promoter-neither. Merged H3K27ac peaks were annotated using HOMER's annotatePeaks.pl (version 4.11) (Heinz et al., 2010). H3K27ac HiChIP 1D peaks were overlapped with ENCODE H3K27ac ChIP-seq peaks obtained from MACS narrowPeak files from primary tissue samples with accession numbers listed in Supplementary Table 8. Number of interacting gene promoters with H3K27ac peaks and number of genes skipped by loops were determined using GenomicRanges' findOverlaps function (version 1.42.0) with gene promoters obtained from TxDb.Hsapiens.UCSC.hg38.knownGene (version 3.10.0).

Comparison to HiChIPdb loops

10kb resolution FitHiChIP loops from H3K27ac HiChIP experiments were downloaded from HiChIPdb (Zeng et al., 2023). hg19 coordinates were converted to hg38 using the easyLiftOver function from the R package easyLift (https://github.com/caleblareau/easyLift, version 0.2.1). Loop sets were converted to GenomicInteractions format in R (version 1.24.0) and the intersection between HiChIPdb loops and our loop set was determined using GenomicRanges' findOverlaps function (version 1.42.0).

Interaction matrix visualization

2D interaction matrices were visualized using Juicebox (version 1.11.08) or with the plotgardener package in R (version 1.2.10) (Kramer et al., 2022).

Eigenvector calculation and A/B compartment annotation

The eigenvector (first principal component of the Pearson's matrix) for H3K27ac HiChiP observed/expected interaction matrices was obtained from .hic files using juicer_tools eigenvector function (version 1.9.9) at 500 kb resolution with KR normalization. The sign of the eigenvector and A/B compartment annotation was assigned based on correlation with DNA methylation eigenvector and compartment analysis obtained from Additional File 2 of Fortin and Hansen, 2015 (Fortin and Hansen, 2015). A positive eigenvector sign is used to indicate A (open) compartment and negative sign to indicate B (closed) compartment, the opposite of Fortin & Hansen, 2015, and thus the eigenvector sign is flipped relative to the sign in Fortin & Hansen, 2015. For hierarchical clustering in Figure 1f, we used CALDER (Liu et al., 2021) (version 2.0) to obtain sub-compartment calls at 10 kb resolution and performed clustering using vectorized sub-compartment annotations based on the compartment rank annotation returned by CALDER.

H3K27ac 1D signal and virtual 4C visualization

One dimensional H3K27ac enrichment and ATAC-seq signal was visualized following normalization by reads in TSS regions as described in the ArchR package (Granja et al., 2020). ATAC-seq signal tracks were obtained from the GDC publication page (Corces et al., 2018). H3K27ac ChIP-seq signal tracks were obtained from ENCODE (accessions ENCFF905FLR and ENCFF873MWG) (Dunham et al., 2012; Luo et al., 2020). Virtual 4C plots were generated from dumped matrices generated with Juicer Tools (1.9.9). The Juicer Tools tools dump command was used to extract the chromosome of interest from the .hic file. The interaction profile of a 10-kb bin containing the anchor was then plotted in R (v.4.0.3) after normalization by the total number of valid read pairs and smoothing with the rollmean function from the zoo package (v.1.8-9).

Generation of union H3K27ac peak and interaction count matrices

One dimensional H3K27ac peaks called by MACS2 were merged using bedtools merge and peak signal calculated using bedtools coverage using one-dimensional H3K27ac signal bed files (v2.28.0). Significant HiChIP interactions identified by FitHiChIP were merged using FitHiChIP's CombineNearbyInteraction.py and loop signal calculated using pgltools coverage (version 2.2.0) (Greenwald et al., 2017). Raw peak and loop signal was normalized using DESeq2's size factors normalization obtained using counts (dds,normalized=TRUE) (version 1.30.1) (Love et al., 2014). CNV correction was performed for cases with matching WGS data by dividing normalized signal by ploidy-corrected relative CNV values for peaks or loops overlapping with amplified genomic intervals (relative CNV > 1). Peaks or loops that overlapped genomic intervals with CNV equal to zero or no CNV call were converted to NA values for those samples. For CNV correction of 2D loop signal, the relative CNV value of

each loop anchor was determined and the normalized loop signal divided by the product of the CNV values at the two anchors. Seven samples did not have matched WGS data for CNV correction and were excluded from further analysis.

Unsupervised hierarchical clustering, cluster purity calculation, and dimensionality reduction
Eigenvectors for H3K27ac HiChIP interaction matrices for chromosomes 1-22 were obtained from .hic files using juicer_tools eigenvector function (version 1.9.9) at 1 Mb resolution with KR normalization and pairwise Pearson correlations calculated using the cor function in R using "pairwise.complete.obs". Heatmap visualization and hierarchical clustering were performed using the pheatmap function in R (version 1.0.12). Clustering assignments were obtained using the cutree function in R with k equal to the number of unique cancer types. Clustering purity and entropy were calculated using the purity and entropy functions from the NMF package in R (version 0.26) (Gaujoux and Seoighe, 2010).

For 1D H3K27ac and loop signal clustering, pairwise Pearson correlations were calculated using the normalized, copy-number corrected count matrices. Peaks and loops on chrX and chrY and those overlapping hg38 blacklist regions (Amemiya et al., 2019) (https://github.com/Boyle-Lab/Blacklist/blob/master/lists/hg38-blacklist.v2.bed.gz) were excluded from analysis. Correlation analysis was performed on reproducible peaks and loops where at least two samples had a normalized count value >=3. Count matrices were log2 transformed using a prior count of 1 to reduce the contribution of variance from elements with low count values and to avoid taking the log of zero. Visualization, clustering and purity calculations were performed as described above. The same count matrices were used for dimensionality reduction and visualization using t-Distributed Stochastic Neighbor Embedding (t-SNE). Log-transformed counts were scaled using Seurat's ScaleData and element counts were ranked by variance using matrixStats rowVars function. The top 10,000 variable elements were used for principal component analysis (PCA) using Seurat's RunPCA function. The top 15 PCs were used for t-SNE dimensionality reduction using Seurat's RunTSNE with perplexity = 5. Samples were colored by cancer type, bulk ATAC-seq cluster annotation (Corces et al., 2018), BRCA subtype (Sanchez-Vega et al., 2018), and ESCA subtype (Cancer Genome Atlas Research Network et al., 2017).

Identification of differential H3K27ac peaks and HiChIP loops by feature binarization
We executed the identification of 'unique' peaks within the HiChIP data, adhering to a predefined methodology. In essence, we log2-transformed the copy number corrected H3K27ac peak count matrix, categorizing individual cancer types as distinct 'groups'. For each peak within the HiChIP peak set, we computed both intragroup mean and standard deviation values. Subsequently, these groups were ranked based on their respective intragroup mean scores. Through an iterative process, we initiated from the second-lowest-ranked group and gauged whether its mean value surpassed the sum of the maximum intragroup mean and the intragroup standard deviation of the subsequent-lower group. This iterative sequence persisted until a group meeting this particular criterion was identified. This point defined the 'breakpoint'. Groups boasting intragroup means exceeding the breakpoint were labeled '1' for that specific peak, while groups situated below the breakpoint received a '0' designation. Peaks lacking a breakpoint were excluded. This 'binarization' process established all '1s' as being greater than any individual '0', thus capturing peaks unique to multiple groups. Combinations present in three or fewer groups were retained. To address multiple hypothesis testing, we devised a contrast matrix for observed combinations and subjected the log-normalized counts matrix to limma's (v.3.38.3) eBayes test. Subsequently, we extracted false discovery rate (FDR)-adjusted P values from differential testing, preserving peaks with FDR values below 0.01. Employing the same aforementioned methodology, we also determined the 'unique' interactions within the HiChIP data by using the log2-transformed copy number corrected H3K27ac interaction count matrix. For motif enrichment analysis, we transformed the 'unique' peaks of each cancer type into the bed format. However, due to the vast genomic span covered by 'unique' interactions, conducting direct motif enrichment analysis proved challenging. As a solution, we intersected the 'unique' interactions per cancer type with the corresponding H3K27ac peaks. Peaks overlapping both anchors were consolidated into the bedgraph format to facilitate motif enrichment analysis. The findMotifsGenome function from HOMER software (v4.11.1) was employed for this purpose, using the parameter '-size given'.

Enhancer rewiring analysis
Using the normalized and copy number corrected consensus FitHiChIP loops from H3K27ac HiChIP 3D data, we intersected the loop anchor with consensus peaks from H3K27ac 1D data as well as promoters of gene transcripts. Promoters are defined as -2500/+250 bp of each TSS using GENCODE v36. In situations where the peak is involved in a given peak-promoter interaction in one sample but not called as a peak by H3K27ac 1D in that sample, "0" will be assigned to the peak-promoter interaction for that given sample. We also focused on enhancer-promoter interactions by excluding H3K27ac 1D peaks overlapping any promoters when interacting with another promoter. Overall, from consensus loops with 10kb anchors, we identified 894,776 enhancer-promoter interactions.

Modeling of oncogene expression with copy number and enhancer activity
To determine the relative contributions of copy number and enhancer activity to variability in oncogene expression, we integrated H3K27ac peaks and interactions, WGS ploidy-corrected CNV calls and HTSeq counts from RNA-seq data for annotated gene loci. Samples missing from any of these datasets were excluded from this analysis. RNA-seq raw counts were normalized using DESeq2's size factors normalization obtained using counts (dds,normalized=TRUE) (version 1.26.0). Union H3K27ac peaks within 1 Mb away from annotated gene TSSs that were supported by peak-TSS interaction loops in HiChIP were considered. To account for increased HiChIP read counts due to CNV, read counts of these TSS-associated H3K27ac peaks were normalized to ploidy-corrected copy numbers as follows: CNV-normalized peak count = (DESeq2-normalized peak count) / (ploidy-corrected copy number * 2 + 1). To assess the variability in gene expression, we first filtered on expressed genes defined as genes with more than 10 transcripts per million (TPM) in more than three samples in the RNA-seq dataset. We then used multiple linear regression to model the DESeq2-normalized RNA-seq gene expression values using the formula RNA ~ H3K27ac + CN, where RNA is the DESeq2-normalized RNA-seq gene expression value, H3K27ac represents terms of log2-transformed, scaled and centered 1D H3K27ac counts of peaks associated with the given gene, and CN represents the ploidy-corrected copy number of the gene. For genes with which more than five H3K27ac peaks were associated, log2-transformed, scaled and centered 1D H3K27ac counts were reduced to five principal components using the pca function in R with ncomp = 5, center = TRUE, scale = TRUE. For genes with 5 or less linked H3K27ac peaks, individual peak signal was used as input for RNA expression modeling rather than PCs. Relative importance of model predictors for each gene was quantified with the Lindeman, Merenda, and Gold (LMG) method using the calc.relimp function in R with type = "lmg", rela = FALSE. To analyze the relative importance of H3K27ac HiChIP signal and copy number of oncogenes, we curated a list of oncogenes and possible oncogenes based on previous analysis (Bailey et al., 2018). Log2 transformation of count data was performed as log2 (count + 1) unless specified otherwise.

Sample specific scATAC-seq data analysis
The processed scATAC-seq archR object (v1.0.1) with cell type annotation was downloaded from https://www.synapse.org/. For each sample with matched H3K27ac HiChIP data, we regenerated archR object and re-calcuated chromatin accessibility peaks for each cell population through MACS2 (v2.1.1) under default setting.

HiChIP integration with scATAC-seq
29 samples with matched H3K27ac HiChIP and scATAC-seq data. A minimum number of 110 non-cancer cells were required in each sample to ensure the power of scATAC-seq peak signal detection in the tumor microenvironment, which ends up with 16 samples for integration. For each matched sample, we examined the co-occurence of H3K27ac peaks and scATAC-seq peaks in the anchor regions of promoter-enhancer interactions. The cell type specific promoter-enhancer interaction was identified when (1) the promoter region of the regulated gene had both H3K27ac and scATAC-seq peaks, (2) the enhancer region defined by the HiChIP interactions had H3K27ac peaks but was uniquely accessible in a specific cell type. The cell type shared promoter-enhancer interaction was defined when the promoter or enhancer regions had both H3K27ac and scATAC-seq peaks but were not limited to a specific cell type. The ambiguous promoter-enhancer interaction was defined when both promoter and enhancer region cannot map to any scATAC peaks. To generalize our sample-specific analysis to the broader population, we performed a correlation analysis between the promoter-enhancer interaction signal and the corresponding cell fraction in the tumor microenvironment. We obtained these cell fractions from scATAC-seq and estimated leukocyte fractions from RNA-seq data. The Spearman correlation coefficient (Rho) was calculated for each correlation, and we applied cutoff values of Rho ≥ 0.30 and Rho ≥ 0.25 to filter the results. For validation of H3K27ac HiChIP deconvolution in tumor microenvironment, the RNA-seq derived leukocyte fraction estimation, ImmuneScore and tumor purity estimation were downloaded respectively from the original publication for correlation analysis (Aran et al., 2015; Thorsson et al., 2018; Yoshihara et al., 2013).

HiChIP integration with cancer associated SNP sites
Cancer-associated SNP data were retrieved from the database available at https://www.ebi.ac.uk/gwas/. We augmented the SNP list by incorporating SNPs in high Linkage Disequilibrium (LD) with GWAS lead SNPs (LD r2 > 0.8). This LD data was sourced from the haploreg website (http://archive.broadinstitute.org/mammals/haploreg/data/). To identify potential regulatory elements associated with these SNPs, we performed an intersection analysis with enhancer peaks. The enhancer peaks were obtained from malignant cell-specific promoter-enhancer interactions, as determined through our prior HiChIP decomposition analysis. This approach allowed us to pinpoint genomic positions where cancer-associated SNPs coincided with enhancer elements.

Identification of non-coding mutation involved H3K27ac modification
62 samples with matched H3K27ac HiChIP and WGS data. We used the somatic mutation calling from WGS data as the ground truth. The mutation allele frequency of H3K27ac HiChIP data was generated using bcfools. First, the global aligned H3K27ac bam files from the FitHiChIP pipeline were piled up through mpileup function from bctools (v1.17). Then, the derived bcf files were converted into vcf files through call function from bcftools. The allele frequency of each somatic mutation was quantified from the vcf files accordingly. The reads coverage of H3K27ac HiChIP at the somatic mutation site was calculated through multiBamSummary from deeptools. The mutation with at least 30 H3K27ac reads coverage was taken as confident calls. The significance of the mutant allele was estimated using Fisher's Exact Test, followed by Benjamini-Hochberg (BH) method for multiple comparison correction.
The H3K27ac signal change involved in the mutation site was quantified using the 2kb window that centered at the mutation position. The 2kb window was splitted into 20bins with each bin equal to 100 bp. The H3K27ac HiChIP signal was calculated through multiBamSummary from deeptools (v2.0) and normalized by the library size and copy number. For each mutation, we performed T test between mutant samples and wild type samples to quantify the difference of CNV corrected H3K27ac signals. To perform multiple comparison correction, we utilized the Benjamini-Hochberg (BH) method.

Quantification of non-coding mutation involved motif enrichment changes
chromVARmotifs R package (v0.2) was used for collection of human transcription factor binding motifs. motifmatchr R package was used for performing motif enrichment analysis. First, a 21 bp sequence centered at mutation position was derived. Then, matchMotifs function was applied to the 21bp sequences from mutant and wildtype for motif enrichment calculation under parameter out="positions" with a p value cutoff 0.01.

AmpliconArchitect reconstruction of complex structural rearrangements
We collected 120 tumor WGS samples from 16 distinct cancer types and 123 matched normal WGS samples from TCGA, all aligned to GRCh38. We ran AmpliconSuite version 0.931.4 (https://github.com/AmpliconSuite/AmpliconSuite-pipeline) which invoked CNVkit (Talevich et al., 2016) to call genome-wide copy number (CN) profiles and identify seed amplicon intervals with CN values larger than 4.5 from these aligned WGS samples. We then ran AmpliconArchitect (AA) (Deshpande et al., 2019) version 1.3_r1 to infer the structure of focal amplifications from each sample, with the aligned WGS reads and seed amplicon intervals as input. A focal amplification is composed of a collection of genomic segments connected by breakpoints indicating either a CN change between two consecutive segments, or a rearrangement connecting two nonadjacent segments. A single sample can contain multiple non-overlapping focal amplifications. AA represents focal amplifications in the form of a copy-number aware breakpoint graph, where nodes represent genome segments and edges represent junctions between segments, including breakpoint connections. AA further decomposes the breakpoint graph into a collection of cyclic and non-cyclic paths, each representing a potential structure or substructure (i..e, local assembly) comprised of genome segments connected by a chain of breakpoints. The structural signatures in these paths are subsequently used to classify the type of focal amplification. Note that AA was run with parameters -insert_sdevs 9 to filter artifactual discordant reads and improve runtime performance, and default parameters otherwise.

Amplicon classification
We ran AmpliconClassifier version 0.4.10 (https://github.com/AmpliconSuite/AmpliconClassifier) using the AA-derived breakpoint graph and cycles files to classify each focal amplification into five categories: (1) cyclic amplification (potential ecDNAs); (2) BFB amplification; (3) Complex non cyclic amplification; (4) Linear amplification; and (5) Invalid focal amplification. We summarize the AmpliconClassifer rules (originally described in Kim et al. and Luebeck et al. (Kim et al., 2020; Luebeck et al., 2023)) as follows. As a prerequisite, focal amplifications must contain ≥10 kb of total genomic segments amplified to at least 5 copies above median ploidy to be considered valid. Focal amplifications were classified as BFB if they met the criteria for a BFB amplification (i.e., if breakpoints representing foldback events account for at least 25% of all SVs in the amplicon, and the cycles containing a foldback account for at least 60% of the length-weighted total CN of valid amplicon paths decomposed by AA). Focal amplifications not classified as BFB were classified as cyclic if there exists a cycle in the breakpoint graph (representing a potential ecDNA structure), and the total copy counts from cycles account for at least 12% of the total length-weighted CN. Acyclic focal amplifications were classified as complex non cyclic if they contained at least 5 breakpoint edges representing rearrangements, suggesting higher-order rearrangements beyond simple indel SV events. All other valid acyclic focal amplifications were classified as linear. We then hierarchically classified samples based on which type of focal amplifications were present in the sample, giving precedence to cyclic, followed by BFB, complex and linear. For example, a sample with both cyclic and complex focal amplifications would be classified as cyclic. Samples without any valid focal amplifications were similarly classified as 'no focal somatic CN amplification detected'.

HiChIP visualization at structural rearrangements with NeoLoopFinder

We ran NeoLoopFinder (Wang et al., 2021) version 0.2.5 to search for chromatin loops on rearranged genomes (corresponding to local assemblies of linked breakpoints) and CN-corrected H3K27ac HiChIP matrices. NeoLoopFinder, by default, computes a genome-wide CN profile and a collection of CN segments from an input HiChIP matrix, and then balances the matrix with a modified ICE procedure by taking the CN segments as input. Input cool files were generated at 10kb resolution from .hic files using HiCExplorer's hicConvertFormat (version 2.2) and balanced using cooler balance (version 0.9.1). We provided the NeoLoopFinder pipeline with CN segments estimated from the corresponding WGS samples (based on ASCAT CNV calls) as its input of the CN-aware matrix balancing procedure with NeoLoopFinder's correct-cnv. Given a list of candidate SVs (potentially from other sources, e.g., WGS or OM), NeoLoopFinder then reconstructs local assemblies representing a chain of one or more SVs from the input list, by shifting or flipping the submatrices according to the coordinates and orientations of the SVs. Therefore, we supplied NeoLoopFinder with a collection of SV breakpoints identified by BRASS from WGS data, which were filtered and used for complex SV assembly with NeoLoopFinder's assemble-complexSVs. In case NeoLoopFinder missed true assemblies, we additionally augmented the assemblies constructed by NeoLooFinder with the collection of local assemblies from AA cycle decomposition as follows. Because NeoLoopFinder does not accept assemblies with duplicated segments, we broke each cycle returned by AA into all possible longest paths of at least 2 non-overlapping segments. We provided these paths as input to NeoLoopFinder to search for chromatin loops in addition to the local assemblies constructed above using neoloop-caller -O neo-loops.txt allValidPairs.cool --assembly assemblies.txt --balance-type CNV --protocol insitu --prob 0.95 --nproc 20. The output of NeoLoopFinder consists of two types of interactions: 'loops,' which represent interactions on a single genomic segment, and 'neo-loops,' representing interactions on two different genomic segments, brought together by an SV. We postprocessed the loops and neoloops identified by NeoLoopFinder in each HiChIP sample (as case sample) by filtering out those that also occur in any other samples without focal amplifications on the same genomic segments (as control samples). In control samples, loops were searched on the same collection of local assemblies as used in the case sample. For comparing the number of loops per classification type, we dropped focal amplifications with total size less than 500kb, which often lead to unreliable classifications, as well as insufficient number of neighboring bins for loop finding.

Co-amplification frequency analysis across TCGA WGS
To identify potential enhancer regions co-focally-amplified with an oncogene of interest (for example, amplified on ecDNA), we binned each focally amplified genome with 10kb resolution in accordance with HiChIP, and counted the number of samples co-amplified with the given oncogene per bin. Due to small cohort size (243 samples in total), the oncogene of interest is often amplified in very few samples. We overcome this limitation by counting, in each 10kb bin, the number of samples co-amplified with the given oncogene within a larger cohort of 1538 WGS samples from Kim et al (Kim et al., 2020). We computed an empirical P-value of co-amplification for each 10kb bin connected with the given oncogene by a loop or neoloop as follows: Let n0 be the number of samples where bin bi is co-amplified with gene g. To compute an empirical permutation based p-value, we generated 10,000 datasets randomly shuffling the focally amplified bins in each sample, such that (i) the distance between the first and last amplified bins after shuffling is at most that distance in the original amplification; (ii) the number of contiguously amplified intervals after shuffling remains the same as the number in the original amplification; and (iii) bins involving g are always amplified. The empirical p-value was given by the fraction of times bin bi was co-amplified with g in at least n0 samples. Finally, empirical P-values were adjusted for multiple comparisons using the Benjamini-Hochberg procedure.

Code Availability
Custom code used in this study is available at https://github.com/NCICCGPO/HiChIP-Manuscript.

For manuscripts utilizing custom algorithms or software that are central to the research but not yet described in published literature, software must be made available to editors and reviewers. We strongly encourage code deposition in a community repository (e.g. GitHub). See the Nature Portfolio guidelines for submitting code & software for further information.

Data

Policy information about availability of data

All manuscripts must include a data availability statement. This statement should provide the following information, where applicable:

- Accession codes, unique identifiers, or web links for publicly available datasets
- A description of any restrictions on data availability
- For clinical datasets or third party data, please ensure that the statement adheres to our policy

Processed data not provided in the supplementary data files is available through the TCGA Publication Page (https://gdc.cancer.gov/about-data/publications/TCGA-HiChIP-2024). Raw HiChIP data as fastq or aligned bam files are available through the NIH Genomic Data Commons portal (https://portal.gdc.cancer.gov/).
The processed RNA-seq, Genome-Wide SNP Array, DNA-methylation, and ATAC-seq data were downloaded from https://portal.gdc.cancer.gov/.
The processed scATAC-seq archR object (v1.0.1) with cell type annotation was downloaded from https://www.synapse.org/.
Cancer-associated SNP data were retrieved from the database available at https://www.ebi.ac.uk/gwas/. The LD data was sourced from the haploreg website (http://archive.broadinstitute.org/mammals/haploreg/data/).

Research involving human participants, their data, or biological material

Policy information about studies with human participants or human data. See also policy information about sex, gender (identity/presentation), and sexual orientation and race, ethnicity and racism.

Reporting on sex and gender	Included in Supplementary Table 1.
Reporting on race, ethnicity, or other socially relevant groupings	Included in Supplementary Table 1.
Population characteristics	Samples were selected from the set of samples previously profiled by bulk ATAC-seq to span the 16 cancer types profiled in this manuscript with a focus on breast cancer and at least 3 samples for each other cancer. Within breast cancer, 3 samples were selected from each major breast cancer subtype (Basal, HER2, LumA, LumB). Samples were prioritized for selection

based on high data quality in previous bulk ATAC-seq experiments and the availability of sufficient nuclei in cryopreserved stocks. Other inclusion or exclusion criteria were not assessed. Additional characteristics including participant age are available at https://portal.gdc.cancer.gov/

Recruitment	All recruitment was done by The Cancer Genome Atlas.
Ethics oversight	This study complied with all relevant ethical regulations and ethical guidance was overseen by the TCGA Program Office. Each study site that contributed biological material had its own ethics board approval. TCGA ethics policies are available at https://www.cancer.gov/ccg/research/genome-sequencing/tcga/history/ethics-policies

Note that full information on the approval of the study protocol must also be provided in the manuscript.

Field-specific reporting

Please select the one below that is the best fit for your research. If you are not sure, read the appropriate sections before making your selection.

☒ Life sciences ☐ Behavioural & social sciences ☐ Ecological, evolutionary & environmental sciences

For a reference copy of the document with all sections, see nature.com/documents/nr-reporting-summary-flat.pdf

Life sciences study design

All studies must disclose on these points even when the disclosure is negative.

Sample size	Samples were selected from the set of samples previously profiled by bulk ATAC-seq to span the 16 cancer types profiled in this manuscript with a focus on breast cancer and at least 3 samples for each other cancer. Within breast cancer, 3 samples were selected from each major breast cancer subtype (Basal, HER2, LumA, LumB). Samples were prioritized for selection based on high data quality in previous bulk ATAC-seq experiments and the availability of sufficient nuclei in cryopreserved stocks. Other inclusion or exclusion criteria were not assessed.
Data exclusions	No data were excluded.
Replication	Replication was not performed due to limited availability of biological material and input requirements for HiChIP library generation.
Randomization	No randomization was done to allocate samples into experimental groups.
Blinding	The samples we analyzed were deidentified by The Cancer Genome Atlas. The investigators were not blinded to group allocation during experiments and outcome assessment.

Reporting for specific materials, systems and methods

We require information from authors about some types of materials, experimental systems and methods used in many studies. Here, indicate whether each material, system or method listed is relevant to your study. If you are not sure if a list item applies to your research, read the appropriate section before selecting a response.

Materials & experimental systems

n/a	Involved in the study	
☐	☒	Antibodies
☒	☐	Eukaryotic cell lines
☒	☐	Palaeontology and archaeology
☒	☐	Animals and other organisms
☒	☐	Clinical data
☒	☐	Dual use research of concern
☒	☐	Plants

Methods

n/a	Involved in the study	
☒	☐	ChIP-seq
☒	☐	Flow cytometry
☒	☐	MRI-based neuroimaging

Antibodies

Antibodies used	All HiChIP was performed using H3K27ac as the target (Abcam ab4729). 2 ug of H3K27ac antibody was used per sample with one million cells as input.
Validation	The antibodies are validated for use on human samples on the manufacturer's websites and give highly reproducible results with published positive control data-sets including ChIP-seq data from primary tissues and ATAC-seq from matched tumor samples.

Plants

Seed stocks	*Report on the source of all seed stocks or other plant material used. If applicable, state the seed stock centre and catalogue number. If plant specimens were collected from the field, describe the collection location, date and sampling procedures.*
Novel plant genotypes	*Describe the methods by which all novel plant genotypes were produced. This includes those generated by transgenic approaches, gene editing, chemical/radiation-based mutagenesis and hybridization. For transgenic lines, describe the transformation method, the number of independent lines analyzed and the generation upon which experiments were performed. For gene-edited lines, describe the editor used, the endogenous sequence targeted for editing, the targeting guide RNA sequence (if applicable) and how the editor was applied.*
Authentication	*Describe any authentication procedures for each seed stock used or novel genotype generated. Describe any experiments used to assess the effect of a mutation and, where applicable, how potential secondary effects (e.g. second site T-DNA insertions, mosiacism, off-target gene editing) were examined.*
